# Vaccine-Preventable Disease Outbreaks Among Healthcare Workers: A Scoping Review

**DOI:** 10.1093/cid/ciae209

**Published:** 2024-04-17

**Authors:** Tasnim Hasan, Michelle Lynch, Catherine King, Charbel Wehbe, Martin Plymoth, Md Saiful Islam, Theodore Iannuzzi, Aiken Dao, Jana Lai, Alexandra Martiniuk, Shalini Desai, Meru Sheel

**Affiliations:** Sydney Medical School, Faculty of Medicine and Health, University of Sydney, Sydney, Australia; Blacktown Hospital, Western Sydney Local Health District, Sydney, Australia; Sydney Infectious Disease Institute, Faculty of Medicine and Health, University of Sydney, Sydney, Australia; School of Public Health, Faculty of Medicine and Health, The University of Sydney, Sydney, Australia; Sydney Infectious Disease Institute, Faculty of Medicine and Health, University of Sydney, Sydney, Australia; School of Public Health, Faculty of Medicine and Health, The University of Sydney, Sydney, Australia; National Centre for Immunisation Research and Surveillance, The Children's Hospital at Westmead, Westmead, Australia; Blacktown Hospital, Western Sydney Local Health District, Sydney, Australia; Blacktown Hospital, Western Sydney Local Health District, Sydney, Australia; School of Population Health, University of New South Wales, Sydney, Australia; Blacktown Hospital, Western Sydney Local Health District, Sydney, Australia; Sydney Medical School, Faculty of Medicine and Health, University of Sydney, Sydney, Australia; Sydney Infectious Disease Institute, Faculty of Medicine and Health, University of Sydney, Sydney, Australia; School of Public Health, Faculty of Medicine and Health, The University of Sydney, Sydney, Australia; National Centre for Immunisation Research and Surveillance, The Children's Hospital at Westmead, Westmead, Australia; Sydney Medical School, Faculty of Medicine and Health, University of Sydney, Sydney, Australia; School of Public Health, Faculty of Medicine and Health, The University of Sydney, Sydney, Australia; Dalla Lana School of Public Health, University of Toronto, Toronto, Ontario, Canada; Immunization, Vaccines and Biologicals Department, World Health Organization, Geneva, Switzerland; Sydney Infectious Disease Institute, Faculty of Medicine and Health, University of Sydney, Sydney, Australia; School of Public Health, Faculty of Medicine and Health, The University of Sydney, Sydney, Australia

**Keywords:** immunization, communicable diseases, health personnel, disease outbreaks, epidemics

## Abstract

**Background:**

Outbreaks of vaccine-preventable diseases (VPDs) in healthcare workers (HCWs) can result in morbidity and mortality and cause significant disruptions to healthcare services, patients, and visitors as well as an added burden on the healthcare system. This scoping review aimed to describe the epidemiology of VPD outbreaks in HCWs caused by diseases that are prevented by the 10 vaccines recommended by the World Health Organization for HCWs.

**Methods:**

In April 2022, CINAHL, MEDLINE, Global Health, and EMBASE were searched for all articles reporting on VPD outbreaks in HCWs since the year 2000. Articles were included regardless of language and study type. Clinical and epidemiological characteristics of VPD outbreaks were described.

**Results:**

Our search found 9363 articles, of which 216 met the inclusion criteria. Studies describing 6 of the 10 VPDs were found: influenza, measles, varicella, tuberculosis, pertussis, and rubella. Most articles (93%) were from high- and upper-middle-income countries. While most outbreaks occurred in hospitals, several influenza outbreaks were reported in long-term-care facilities. Based on available data, vaccination rates among HCWs were rarely reported.

**Conclusions:**

We describe several VPD outbreaks in HCWs from 2000 to April 2022. The review emphasizes the need to understand the factors influencing outbreaks in HCWs and highlights the importance of vaccination among HCWs.

## Teaser

This scoping review identified all published outbreaks of vaccine-preventable diseases in healthcare workers between 2000 and 2022. Vaccine-preventable diseases were the 10 diseases for which vaccines are recommended for healthcare workers by the World Health Organization.

## Graphical Abstract

**Graphical Abstract:**
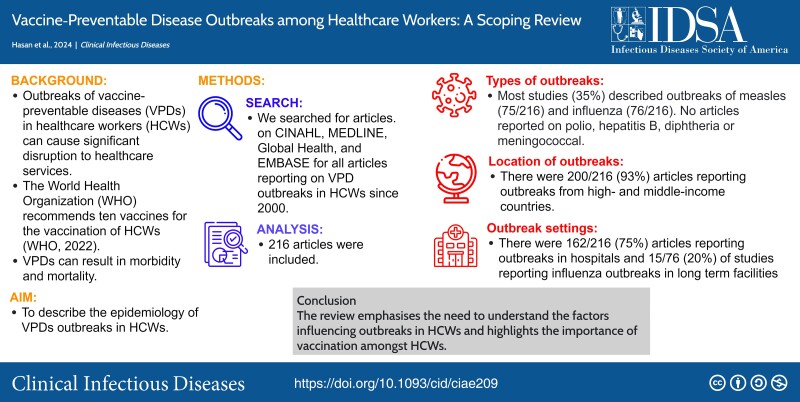
This graphical abstract is also available at Tidbit: https://tidbitapp.io/tidbits/vaccine-preventable-disease-outbreaks-among-healthcare-workers-a-scoping-review-678ca9e3-7ab1-4ece-b3e0-f31099ce6a52

The coronavirus disease 2019 (COVID-19) pandemic highlighted and reinforced the impact that disease outbreaks among healthcare workers (HCWs) can have on health systems. Healthcare workers are those who are engaged in any form of employment that is aimed at improving health [1]. Due to the increased risk of transmission in healthcare settings [1], investigating and preventing outbreaks of vaccine-preventable diseases (VPDs) among HCWs is crucial to ensure the safety of HCWs, patients, and the broader community. The World Health Organization (WHO) recommends that HCWs be vaccinated as per their countries’ vaccination schedule and consideration be given as appropriate to vaccinate against the following key outbreak-prone VPDs: tuberculosis (TB), hepatitis B virus (HBV), poliomyelitis, diphtheria, measles, rubella, meningococcal disease, influenza, varicella, and pertussis [1] (Table 1). COVID-19 was added to this list in 2022 [1].

**Table 1.** Vaccine Antigen by Vaccine-Preventable Disease and WHO Recommendations for Healthcare Workers

Organism	Vaccine Antigen	WHO Recommendations for Vaccination of HCWs
*Bordetella pertussis*	Pertussis	Recommended for all HCWs
Poliovirus	Polio	Recommended for all HCWs
Measles virus	Measles	Recommended for all HCWs
Severe acute respiratory syndrome coronavirus 2	COVID-19	Recommended for all HCWs
Rubella virus	Rubella	Recommended for all HCWs where it is available
Influenza virus	Influenza	Annual vaccination recommended for all HCWs
*Mycobacterium tuberculosis*	BCG	Recommended for HCWs at risk of exposure in low- and high-TB-incidence areas
*Corynebacterium diphtheriae*	Diphtheria	Recommended for HCWs at risk of exposure
*Neisseria meningitidis*	Meningococcus	Recommended every 3–5 y for HCWs at continued risk of exposure
Hepatitis B virus	Hepatitis B	Recommended for HCWs working in exposure-prone practices (eg those exposed to blood and blood products)
Varicella-zoster virus	Varicella	Consider vaccination of HCWs

Data from reference [1].

Abbreviations: BCG, Bacillus Calmette-Guérin; COVID-19, coronavirus disease 2019; HCW, healthcare worker; TB, tuberculosis; VPD, vaccine-preventable disease; WHO, World Health Organization.

Vaccinating HCWs is one means to provide direct protection to a population who have prolonged, close contact with patients. While healthcare acquisition of VPDs can be moderated through robust organizational and national policies focused on HCW vaccination and strict infection-control procedures, outbreaks of VPDs in HCWs continue to be reported [1, 2]. Outbreaks can result in increased morbidity and mortality in both HCWs and patients, increased healthcare costs, and HCW absenteeism [2, 3]. Vaccination of HCWs is an effective means by which healthcare-associated infections of VPDs in HCWs can be prevented [3–5]. Despite this, vaccination rates of HCWs are often suboptimal due to several factors, including access barriers to vaccinations, lack of vaccination-specific policy recommendations, low uptake of vaccines by HCWs, and vaccine hesitancy [2]. These factors emphasize the need to broaden the understanding of VPD outbreaks among HCWs to improve policies that aim to increase vaccination uptake by HCWs.

In this scoping review we aimed to describe the epidemiology of VPD outbreaks in HCWs to examine transmission factors including vaccination. We focused on VPDs that can be prevented by the 10 vaccines recommended for HCWs by the WHO with the goal of informing vaccination policies for HCWs and the prevention of VPDs in healthcare settings.

## METHODS

This scoping review was conducted using the Preferred Reporting Items for Systematic Reviews and Meta-Analyses extension for Scoping Reviews (PRISMA-ScR). A protocol was developed according to the PRISMA-ScR guidelines and registered on Open Science Framework (10.17605/OSF.IO/VMKJR) (Supplement 1). This scoping review aimed to identify VPD outbreaks in HCWs globally, divided by income group of a country (using World Bank classification), to identify vaccination status of HCWs and to examine differences in outbreaks in health workers employed in healthcare settings and non-healthcare settings.

### Search Strategy and Data Sources

We searched Ovid MEDLINE (1946–7 April 2022), Ovid EMBASE (1974–5 April 2022), CINAHL Complete via EBSCO (1937–April 2022), and Global Health via Ovid SP (1910–Week 14, 2022) for literature on VPD outbreaks in HCWs from January 2000 to 8 April 2022. The search was limited to articles published from the year 2000 as vaccination programs have evolved over recent decades and to maintain relevance to contemporary policies and VPD outbreak epidemiology in HCWs. The search terms covered 3 domains: outbreak terminology, the 10 WHO-recommended VPDs, and HCW terms. The full Ovid MEDLINE search strategy is available in Supplement 2. Reference lists of included full texts were screened to identify any additional papers. Content experts were approached to identify relevant gray literature.

### Inclusion Criteria

Studies were included if they reported an outbreak in HCWs of any of the 10 VPDs with WHO recommendations for vaccinations for HCWs. As the definition of an outbreak varies by disease and setting, studies describing any number of cases above what was normally expected or described to be an outbreak were included. Healthcare workers included all those defined by WHO as involved in patient care, such as clinicians, laboratory staff, administrative staff, health-setting cleaners, and community health workers [1]. Articles published in all languages were included, with translation performed using those fluent in the language or via Google Translate (https://translate.google.com/) or ChatGPT (https://chatopenai.com). All study types were included, including studies reporting quantitative or qualitative data.

### Exclusion Criteria

Articles were excluded for the following reasons: were published prior to 2000 or the outbreak occurred before 2000, were conference abstracts, did not report outbreaks in HCWs, and if there were insufficient data for extraction. Studies solely reporting COVID-19 were excluded as several reviews reporting outbreaks of COVID-19 in HCWs already existed, including 1 with 28 studies describing 120 000 HCWs [6].

### Study Selection and Screening

All citations were exported to EndNote (Clarivate, USA, version 20) for automatic and manual deduplication. All titles, abstracts, and full texts were independently reviewed by 3 authors (M. L., M. S. I., J. L.) using COVIDence (https://www.covidence.org). Conflicts were resolved through discussion between reviewers or through consultation with a third author (J. L., M. S., C. K.).

### Data Extraction

For each included article the following information was extracted about each outbreak: country(ies) and setting; disease, year(s) and duration; occupation of affected HCWs; reported epidemiological parameters (eg, number of cases, attack rate); age and sex of affected HCWs; presumed index case; origin (nosocomial/community-acquired); disease outcome in HCWs (infection, hospitalization, mortality); public health response including outbreak-response immunization; pre-outbreak vaccination status of affected and overall HCWs; onward transmission from HCWs to patients; strategies for improving vaccination; factors contributing to the outbreak; and study recommendations reported by authors. Microsoft Excel (Microsoft Corporation, Redmond, WA, USA) was used for data extraction.

### Data Analysis

Descriptive statistics were used to summarize quantitative data for each of the VPDs. A qualitative synthesis on factors contributing to outbreaks and other public health determinants was performed including country data, which were used to classify countries by WHO region and World Bank income group, information about outbreak management, infection control and management.

## RESULTS

### Search Results

**Figure 1.**
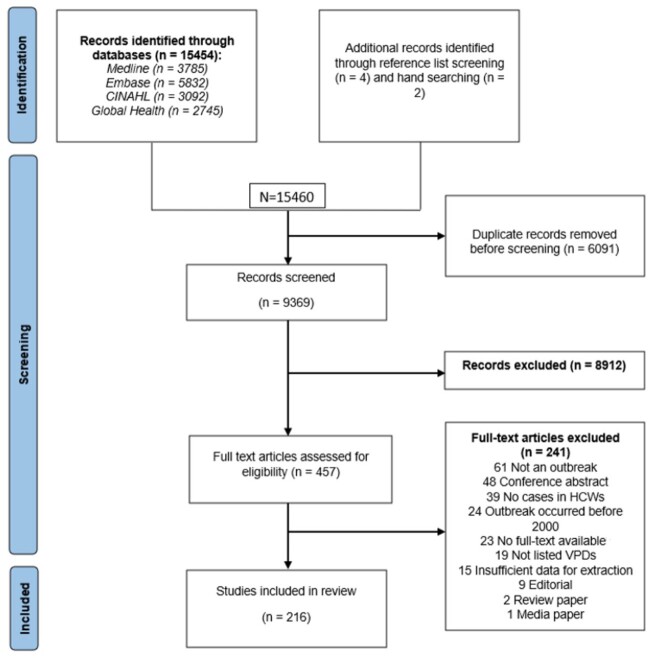
PRISMA (Preferred Reporting Items for Systematic Reviews and Meta-Analyses) 2009 flowchart detailing the selection process for articles on VPD outbreaks among healthcare workers into the review. Abbreviations: HCW, healthcare worker; VPD, vaccine-preventable disease.

A total of 9363 distinct articles were identified from the search strategy (Figure 1). Following title and abstract screening, 457 articles were identified for full-text screening, of which 216 were included in the final review. Twenty-one (10%) non–English-language articles were included (7 French, 6 Japanese, 3 Spanish, 2 Czech, 1 Mandarin, 1 Portuguese, 1 Slovenian).

### Study Characteristics

Table 2 displays the key characteristics of studies included in the review by VPDs. Most studies were cohort studies (90%). There were 3 (1%) cross-sectional studies, 3 (1%) case-control studies, 10 (5%) case series, and 7 (3%) case reports included in this review. Details of individual studies can be found in Supplementary Tables 3–9. No studies met the inclusion criteria for meningococcal disease, polio, HBV, or diphtheria.

**Table 2.** Characteristics of Articles Describing Vaccine-Preventable Disease Outbreaks in Healthcare Workers, 2000–2022

	No. of Articles Included	Studies From HICs or UMICs,^a^ n (%)	Studies With Outbreaks Occurring in Hospitals, n (%)	Studies Reporting Outbreaks With Nosocomial Origin, n (%)	Overall Reported HCWs Vaccinated Pre-Outbreak,^b^ n/N (%)	Studies Reporting HCW as Index Case, n (%)	Studies Reporting Transmission From HCW to Patient(s), n (%)
Influenza	76	71 (97%)	51 (67%)	52 (68%)	473/5154 (9%): 2992 ILI, 2162 confirmed	8 (11%)	11 (14%)
Measles	75	73 (97%)	57 (76%)	14 (19%)	125/1724 (7%): 330 partially vaccinated	6 (8%)	17 (23%)
Varicella	16	8 (50%)	15 (94%)	12 (75%)	7/78 (9%)	2 (13%)	0 (0%)
Tuberculosis	25	25 (100%)	19 (76%)	22 (88%)	NA; total cases: 277 TB disease, 324 TB infection	5 (20%)	2 (8%)
Pertussis	22	22 (100%)	18 (82%)	21 (95%)	10/251 (4%)	13 (59%)	9 (41%)
Rubella	2	1 (50%)	2 (100%)	2 (100%)	9/38 (24%)	1 (50%)	1 (50%)

Abbreviations: HCW, healthcare worker; HIC, high-income country; ILI, influenza-like illness; NA, not available; TB, tuberculosis; UMIC, upper-middle-income country; VPD, vaccine-preventable disease.

^a^Income status of country according to the World Bank: https://datatopics.worldbank.org/world-development-indicators/the-world-by-income-and-region.html.

^b^Many studies did not report vaccination coverage in HCWs; where the percentage vaccinated was reported, the approximate number of HCWs vaccinated was calculated using the total number of HCWs in the sample. Vaccination coverage of HCW was reported from healthcare registries, self-reported by HCWs, or determined by serology results.

**Figure 2.**
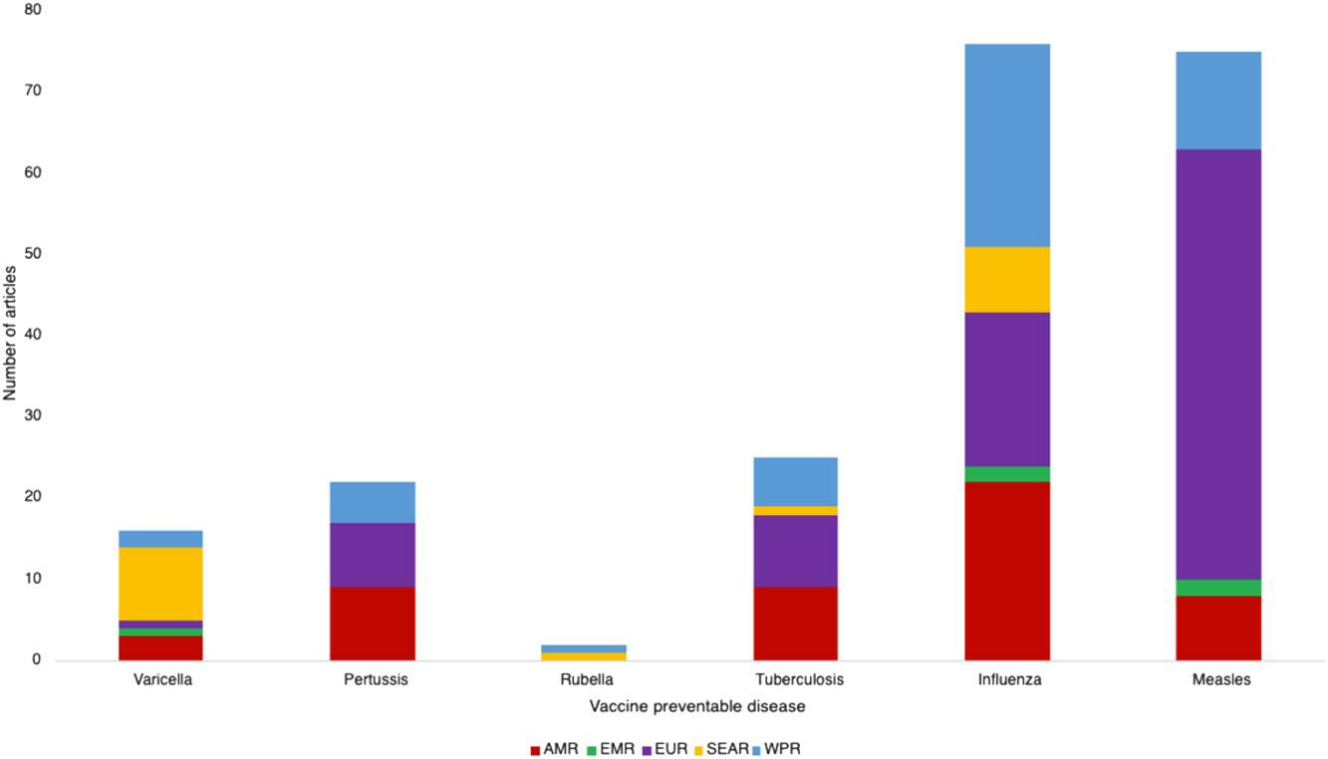
Proportion of articles discussing VPD outbreaks in HCWs, by disease, by WHO region, 2000–2022. Abbreviations: AMR, Region of the Americas; EMR, Eastern Mediterranean region; EUR, European region; HCW healthcare worker; SEAR, South East Asian region; VPD, vaccine-preventable disease; WHO, World Health Organization; WPR, Western Pacific region.

Over 90% (n/N = 193/216) of included studies were from the European region, a region of the Americas, or a Western Pacific region (Figure 2). There were no articles from the African region. Over 90% (n/N = 200/216) of the studies were from high-income or upper-middle-income countries. Sixteen articles (7.4%) were from lower-middle-income settings. No studies were from low-income countries.

Ninety-two (43%) outbreaks lasted 2 months or less, while 44 (20%) articles described outbreaks lasting 6 months or longer. Most outbreaks affected patient-facing HCWs (doctors and nurses) who were females, although exact demographic data on affected HCWs were often missing.

### Outbreak Characteristics

#### Influenza

Seventy-six (35%) articles described influenza outbreaks (Supplementary Table 3). Most (n = 59, 78%) outbreaks occurred during influenza seasons, of which 31 of 59 (53%) occurred during the 2009 influenza pandemic. In 36 (47%) studies, after a laboratory-confirmed case of influenza, influenza-like illness was presumed to represent infection with influenza virus. Fifteen (20%) studies reported outbreaks in long-term-care facilities. Nine (60%) reported higher vaccination coverage among residents compared with HCWs. In both hospitals and care facilities, HCW mortality was reported in only 2 studies [7, 8]; however, 6 (7.9%) studies described mortality among patients or residents due to influenza infection. Factors contributing to outbreaks included HCWs working while sick (n = 7, 9%) and lack of personal protective equipment (PPE) (n = 5, 7%). Outbreak-response strategies included the following: prophylactic oseltamivir (n = 24, 32%), vaccination of contacts (n = 14, 18%), enhanced infection prevention and control (n = 20, 26%), isolation at home (n = 3, 4%), and cancellation of healthcare services (n = 2, 3%).

#### Measles

Seventy-five (35%) studies described measles outbreaks. Fifty (67%) articles described outbreaks in Europe, of which 24 of 50 (48%) reported on clusters of measles cases between 2016 and 2018 (Supplementary Table 4). Seventeen (23%) described the index case to be returning travelers, Roma migrants, or refugees. Thirteen (18%) studies reported hospitalization of HCWs for complications including pneumonia, dehydration, or for the purposes of isolation. Three (4%) studies reported severe infection in HCWs [9–11]. There was no reported HCW-associated mortality. Outbreak response included isolation of cases using airborne precautions or in measles wards (n = 25, 33%), reinforcement of infection control (contact tracing, training, education, and policy development) (n = 50, 67%), and provision of measles immunoglobulin for contacts (n = 9, 12%). Vaccination was recommended as part of the response in 45 (60%) studies. Factors contributing to the outbreak included delays in isolation of cases (n = 5, 7%), low HCW vaccination rate (n = 30, 39%), and uncontrolled community outbreaks (n = 11, 15%).

#### Varicella

Sixteen (7%) articles described varicella outbreaks in HCWs (Supplementary Table 5). Four reported outbreaks in intensive care settings (25%). Interestingly, 2 (13%) outbreaks occurred in autopsy rooms through handling of a deceased infected patient [12, 13]. Seventy-eight varicella cases were reported in HCWs; all recovered without hospitalization and 1 experienced prolonged symptoms [14]. Vaccinated cases were reported to be milder. Outbreaks caused significant disruption to healthcare activities, including the temporary closure of essential services (eg, chemotherapy unit) and healthcare absenteeism. Only 1 article discussed policy implications—universal vaccination for staff and visitors following the outbreak [15]. Vaccination was used as part of outbreak response in 9 (56%) studies. Four (25%) studies reported using acyclovir for postexposure prophylaxis.

#### Tuberculosis

Exposure to TB can cause both latent TB infection and active TB disease. Of the 25 (12%) articles, 17 studies (68%) identified latent TB infection and 10 (40%) identified cases of active TB disease (Supplementary Table 6). One study identified all nosocomial cases of TB reported over a 38-year period [16]. Tuberculosis treatment outcomes for HCWs were often missing, likely due to the prolonged treatment duration. Where TB treatment outcomes were known, all HCWs recovered without complications. One study measured interferon gamma release assay (IGRA) at monthly intervals and found the conversion followed by reversion to negative IGRA test results after an inadvertent laboratory sample exposure [17]. Several active TB cases were described where HCWs were exposed to aerosol-generating procedures, without adequate infection-control procedures [18, 19]. Genomic testing was used to link clusters in 2 separate papers where outbreaks of active TB disease were reported in patients and HCWs; the genomic testing was able to determine that some of the reported HCWs' TB disease was likely unrelated to the described outbreak cluster [20, 21].

#### Pertussis

Twenty-two (10%) studies reported pertussis outbreaks, all in high- or upper-middle-income countries (Supplementary Table 7). Nine (41%) described outbreaks in pediatric or neonatal units (9 studies, 41%), including 4 in neonatal intensive care. Azithromycin or erythromycin prophylaxis was recommended as part of the outbreak response in 16 (73%) studies. Only 5 (23%) studies described vaccination as part of outbreak response, with lack of an available adult vaccine being a barrier to vaccination [22, 23]. Factors contributing to outbreaks included staff not taking time off work despite being unwell (n = 3, 14%), suboptimal PPE use (n = 2, 9%), and lack of immunity in neonates.

#### Rubella

Only 2 (1%) studies reported rubella outbreaks in HCWs (Supplementary Table 8). All infected cases recovered without prolonged illness. Lack of vaccination was considered a significant contributor to the outbreaks, with only 9 (24%) of affected HCWs vaccinated. Both articles discussed vaccination as part of the outbreak response.

## DISCUSSION

This scoping review found 216 studies describing outbreaks of VPDs in HCWs. Almost all studies reported outbreaks in high- and upper-middle-income countries, with the majority of studies describing measles and influenza outbreaks. Reported outbreaks most often occurred in hospitals and affected HCWs engaged in clinical activities. Most HCWs who were infected had an unknown vaccination status for the VPDs they were infected with.

Vaccination coverage of HCWs in the included studies was determined from healthcare registries, serology results, or by self-reporting. In cases where HCW vaccination status was known, uptake was poor, presenting a significant risk for outbreaks of VPDs among HCWs. Consequences of VPD outbreaks include disruption to healthcare services and absenteeism. While HCWs did not experience severe complications, nosocomial infections in patients and residents resulted in morbidity as well as mortality. Despite these risks, there is evidence that vaccination against VPDs among HCWs remains low in many high-income and upper-middle-income settings [24–26]. In the literature assessing HCW attitudes to vaccination, HCWs who were older and non-physicians were more likely to refuse vaccination [24, 26]. While barriers to vaccination were rarely reported in the included studies, other articles have reported barriers to vaccine uptake among HCWs, including lack of clear policies and communication, difficulties with vaccine access, fears of adverse events, and denial of risk [25, 26]. Furthermore, surveys of HCWs have found poor knowledge about vaccination effectiveness, knowledge of which vaccines were recommended, as well as knowledge about one's vaccination status [27]. Further studies and assessments to understand the behavioral and social drivers of vaccination among HCWs are warranted.

The current review found several studies reporting measles outbreaks in HCWs in Europe, which included community and healthcare settings. For example, Portugal reported the first nosocomial measles outbreak after 12 years in 2017 [28]. A resurgence of measles in 47 European countries during 2017–2018 led to more than 80 000 infections, with 72 measles-related deaths [29]. Vaccination coverage in these outbreaks was the most important determinant of the incidence of infection, with vaccination uptake below 84% being a much greater contributor to outbreaks compared with travel, tourism, or migrants [30].

This review did not find any articles on HCW outbreaks of meningococcus, diphtheria, polio, or HBV. The widespread availability of the polio vaccine has resulted in the elimination of endemic wild-type polio in all but 2 countries globally [31]. In contrast, widespread community meningococcal and diphtheria outbreaks have been reported, mostly in lower-middle- or low-income countries [32, 33]. It is possible in such settings that HCW-related outbreaks were not as widely reported. Our search strategy found 4 articles describing HBV outbreaks in HCWs occurring before the year 2000, with HBV spreading from HCW to patients. The lack of more recent studies may be due to policies recommending HBV vaccination in HCWs in high- and middle-income countries, barriers to detection, and improved infection control to reduce occupational exposure [2]. However, a lack of screening and stigmatization of HBV infections [34] may explain the paucity of studies reporting HBV infections in HBV-endemic countries.

### Prevention Strategies and Recommendations

Strategies to improve vaccination include implementation of policies, annual vaccination clinics, easy access to vaccination programs, and education about the risks of being unimmunized or under-immunized [1, 25]. While mandatory vaccination of HCWs may improve vaccine uptake, this can be controversial [1, 25]. Many studies included in our review reported difficulties in obtaining information about HCW vaccination status, suggesting the absence of centralized HCW vaccination registries and screening during recruitment of HCWs. Furthermore, there was limited information about vaccination policies. A survey of 36 European countries found that 35 countries had policies for influenza, 35 for HBV, 28 for measles, and 19 for varicella vaccination for HCWs [2]. The study also found that there was an increase in countries recommending vaccination in HCWs compared with 2011 [2]. A similar study conducted globally by WHO found only 51 of 103 (49.5%) countries had national HCW vaccination policies [35].

Data collected from a survey included in the Joint Reporting Forms reported by member states to WHO suggested that, of the 43 countries with studies included in this scoping review, 25 completed the HCW survey: 17 had national policies and 8 did not have national policies [35] (S. Desai, personal communication, 30 October 2023). Within healthcare settings, it is important to establish a registry of HCWs with their vaccination status, with appropriate policies and procedures to ensure that staff are up to date with vaccination requirements. This should be done within the larger context of occupational health and safety.

The key strengths of this scoping review included a search for 10 VPDs and the inclusion of several articles for each of the 6 different VPDs for which reports of VPD outbreaks in HCWs were found. We also presented quantitative data on HCW infection, attack rates, and vaccination coverage. This study was limited by publication bias; there were limited studies from lower-middle- and low-income countries. There were also no studies from the WHO Africa region. The frequency of outbreaks, how they are investigated, and the impact on HCWs in such settings were therefore difficult to fully determine. While extensive gray literature was not searched, additional reports were sought from experts, but none were identified. Most of the studies lacked data on vaccination status of HCWs, outbreak-response strategies, and demographic details of affected HCWs. Outbreaks of TB are difficult to interpret as the Bacillus Calmette-Guérin (BCG) vaccine is poorly efficacious and mode of transmission can be difficult to ascertain. The TB studies included in this review only included 1 study from a high-TB-burden country and lacked longitudinal TB-reactivation data. Articles included in this review reported outbreaks before the COVID-19 pandemic. While COVID-19 vaccination among HCWs was high [36], the pandemic has otherwise compromised vaccination programs globally [37], with increasing reports of VPD outbreaks globally, including measles [38], and the long-term impacts that the pandemic will have on vaccination uptake and VPD outbreaks is yet to be determined.

This scoping review provides comprehensive insight into outbreaks associated with VPDs in HCWs. A surprising number of VPD outbreaks were reported in high-income and upper-middle-income countries over the last 20 years. The review provides an opportunity for healthcare systems to recognize gaps, better understand the cause of outbreaks, and implement HCW vaccination programs. The latter should include policies, reporting, and documentation of HCW vaccination status and access to vaccines to prevent future outbreaks that can affect HCWs, patients, and visitors in healthcare facilities. Protecting HCWs through vaccination against VPDs would align with and help countries meet goals of life-course vaccination and “leaving no one behind”—in line with strategic priorities of the Immunization Agenda 2030 [39].

## Supplementary Data

Supplementary materials are available at *Clinical Infectious Diseases* online. Consisting of data provided by the authors to benefit the reader, the posted materials are not copyedited and are the sole responsibility of the authors, so questions or comments should be addressed to the corresponding author.

## Supplementary Material

ciae209_Supplementary_Data

## Notes

***Author Contributions.*** Full-text articles were extracted by multiple authors and a second author reviewed extraction of quantitative variables (T. H., M. L., C. W., M. P., T. I., A. D., M. S. I.). A third reviewer resolved conflicts (T. H., M. L., M. S., A. M.).

***Acknowledgments.*** The authors thank Tonmoy Sarkar, S. M. Murshid Hasan, Sazzad Hossain Khan, and Hibah Kolur for their assistance with screening of articles during the early stages of the study. They also thank Cyra Patel for assistance with locating full-text articles. We would like to extend our acknowledgments to provide a special thank you to co-author and friend Dr Jana Lai who sadly passed away while this project was underway. Jana provided substantial contributions to this work and is greatly missed.
