# Supplementary material for: Vaccine-Preventable Disease Outbreaks Among Healthcare Workers: A Scoping Review
Source: Clin Infect Dis. 2024 Apr 17;79(2):555–61. doi: 10.1093/cid/ciae209 (PMC11327795; doi:10.1093/cid/ciae209)
Supplement: ciae209_Supplementary_Data [file ciae209_supplementary_data.zip › Supplemental Material clean.docx]

**Supplemental Material for Vaccine-Preventable Disease Outbreaks among Healthcare Workers: A Scoping Review**

**Supplement 1:** Protocol for Vaccine-Preventable Disease Outbreaks among Healthcare Workers: A Scoping Review as registered on Open Science Framework

**Title: Vaccine-Preventable Disease Outbreaks among Healthcare Workers: A Scoping Review**

**AUTHORS**

Michelle Lynch^1^, Md Saiful Islam^2^, Catherine King^1, 3, 4^, Jana Lai^4^, Alexandra Martiniuk^1^, Phoebe Williams^1^, Shalini Desai^5^, Meru Sheel^1^

**Author affiliations**

^1^ Sydney School of Public Health, Faculty of Medicine and Health, The University of Sydney, Sydney, New South Wales, Australia

^2^ National Centre for Epidemiology and Population Health, ANU College of Health and Medicine, The Australian National University, Canberra, Australian Capital Territory, Australia

^3^ The Children's Hospital at Westmead Clinical School, Faculty of Medicine and Health, The University of Sydney, Sydney, New South Wales, Australia

^4^ National Centre for Immunisation Research and Surveillance, The Children’s Hospital at Westmead, Westmead, New South Wales, Australia

^5^ Department of Immunization, Vaccines and Biologicals, World Health Organization, Geneva, Switzerland

**Contact person:**

Meru Sheel

Email: [meru.sheel@sydney.edu.au](mailto:meru.sheel@sydney.edu.au)

Address: Sydney School of Public Health, Faculty of Medicine and Health, The University of Sydney, Sydney, NSW

**INTRODUCTION**

**Rationale**

Outbreaks of vaccine-preventable diseases (VPDs) among healthcare workers (HCWs) have been reported globally and significantly impact health systems. Investigating VPD outbreaks among HCWs is vital to ensure the safety of HCWs, patients and the broader community. This has become particularly relevant in the context of COVID-19.

The World Health Organisation (WHO) recommends HCWs be vaccinated against several antigens, including BCG, Hepatitis B, Polio, Diphtheria, Measles, Rubella, Meningococcal, Influenza, Varicella and Pertussis (1).

Table 1: *Vaccine Antigen by VPD and Approximate Year of Vaccine Approval or Introduction*

| ***Vaccine Antigen*** (1) | ***VPD*** | ***Approximate Year of Vaccine Approval or Introduction*** (2) |
| --- | --- | --- |
| BCG | Tuberculosis | 1921 |
| Hepatitis B | Hepatitis B | 1981 |
| Polio | Poliomyelitis | 1955 |
| Diphtheria | Diphtheria | 1923 |
| Measles | Measles | 1963 |
| Rubella | Rubella | 1971 |
| Meningococcal | Meningococcal disease | 1974 |
| Influenza | Influenza | 1945 |
| Varicella | Chickenpox | 1995 |
| Pertussis | Whooping cough | 1915 |

A number of potential factors contribute to VPD outbreaks in HCWs. HCWs are regularly in close contact with patients for extended periods of time, increasing their susceptibility to any VPD their patients may be carrying. In some settings, there may be low uptake of vaccines in HCWs due to access barriers, lack of policy recommendations, low compliance, or vaccine hesitancy, contributing to increased risk of VPD outbreaks (3). Vaccine hesitancy in HCWs is particularly concerning due to their influential and trustworthy role within the community (3). These factors emphasise the need to deepen the understanding of VPD outbreaks and barriers to vaccination among HCWs.

Ensuring optimal vaccination coverage and preventing VPD outbreaks can have many benefits. For example, vaccination of HCWs against COVID-19 has resulted in reduced absenteeism, duration of absenteeism and COVID-19 morbidity and mortality, which is expected to safeguard HCWs, services and costs (4). Furthermore, vaccination of HCWs against influenza may reduce costs associated with absenteeism by reducing the incidence of infection and duration of absenteeism (5).

To prevent VPD outbreaks among HCWs and potential spread to the community, it is important to have a better understanding of the nature of the outbreaks, vaccination and transmission factors contributing to these outbreaks. There do not appear to be any systematic or scoping reviews of VPD outbreaks among HCWs. Understanding the epidemiology of VPDs among HCWs and the barriers that prevent effective implementation of HCW vaccination programs, particularly in resource-constrained settings, will contribute to informing policies and future research. While COVID-19 outbreaks in HCWs is beyond the scope of this review, efforts will be made to assess the implications of study findings taking into account the lessons learnt from COVID -19.

**Objectives**

Primary: To describe the epidemiology of VPD outbreaks in HCWs.

Secondary:

1. To assess differences in VPD outbreaks in HCWs in low to middle income countries (LMICs) and high-income countries (HICs).

2. To examine the differences in VPD outbreaks in HCWs in healthcare settings and non-healthcare settings.

3. To identify any barriers to HCW vaccination in the context of the VPD outbreak under study.

**METHODS**

**Protocol and Registration**

The Preferred Reporting Items for Systematic reviews and Meta-Analyses extension for Scoping Reviews (PRISMA-ScR) was used to draft this protocol. The protocol was registered on Open Science Framework on 22/04/2022.

**Eligibility Criteria**

Inclusion Criteria

- Articles published from the year 2000 onwards will be included. This is to specifically target contemporary evidence noting the evolution of vaccination programs in the last 20 years as well as the advancements in infection, prevention, and control measures in healthcare settings.
- Include quantitative and qualitative data on VPD (See table) in HCWs.
  - As the definition of an outbreak varies by disease and setting, studies describing any number of cases above what is normally expected or where the paper describes it to be an outbreak will be included. For example, in the case of polio, one case of wild type polio is often considered an outbreak.
  - HCWs will include all those defined by WHO (2021) as those involved in patient care and public health, such as laboratory staff, administrative and service staff, health setting cleaners, healthcare professionals, epidemiologists, and community health workers.
- Articles published in all languages will be included. Efforts will be made to translate full non-English articles meeting the inclusion criteria using those fluent in the language or via Google Translate ([https://translate.google.com/](https://translate.google.com/).)).
- Data from quantitative, qualitative, and mixed methods studies will be included.
- Only the VPDs as listed in the WHO table as recommended vaccines for HCWs will be included, listed as:
  - BCG; Hepatitis B; Polio; Diphtheria; Measles; Rubella; Meningococcal; Influenza; Varicella; and Pertussis.

Exclusion Criteria

- Reports published before 2000 will be excluded.
- Studies solely describing COVID-19 outbreaks will be excluded for the following reasons:
  - Logistical, human resources and time reasons.
  - COVID-19 became a VPD in 2021, but multiple papers from 2020 were published on COVID-19 in HCWs.
  - Pandemic response immunisation required special planning, under which HCWs were already a priority group (WHO SAGE prioritisation roadmap).
  - Multiple systematic reviews on COVID-19 in HCWs are underway (as per PROSPERO), which will address the knowledge gap.
  - For these reasons, we will exclude COVID-19 but will consider triangulating our findings with systematic reviews published on COVID-19.
- Studies will be excluded if they do not contain sufficient data for extraction.

**Information Sources**

We will search Ovid MEDLINE, CINAHL via EBSCO, Ovid EMBASE and Global Health via Ovid SP. Bibliographies of articles identified for inclusion following the full-text screening will be reviewed for additional relevant papers. Grey literature sources will be hand-searched and identified through consultation with content experts. Google Scholar will be searched for grey literature using key terms for up to 10 pages. Additional sources like the Global Immunization Newsletter, WHO IRIS may be searched for grey literature.

**Search Strategy**

A full electronic search strategy of at least one database will be uploaded as a supplementary file once finalised.

**Selection of sources of evidence**

Screening of the titles and abstracts of items identified in the search will be completed by a minimum of two independent researchers. The titles and abstracts will be screened using the inclusion and exclusion criteria.

Items meeting the inclusion criteria will be included in a full-text review. Disagreements will be resolved by consulting with a third reviewer.

A PRISMA-SCR Flow chart will be used to explain the study selection process.

We will use Covidence ([www.covidence.org](http://www.covidence.org/)) for the processes of screening.

**Data items**

We will extract data on the following variables:

• Study author names

• Study publication year

• Study design

• Countr(ies) of outbreak (areas; cities)

• Year(s) and month(s) of outbreak

• Type of healthcare setting

• Occupation of the HCWs

• Epidemiology parameters of outbreak (e.g., number of cases, incidence)

• Age of HCWs

• Sex of HCWs

• Origin of outbreak (nosocomial / community-acquired)

• Disease outcome in HCWs (infection, hospitalisation, mortality)

• Method of testing for the VPD (VPD Diagnostic tools/process)

• Public health response to the outbreak, including outbreak response immunisation (ORI), administration of post-exposure prophylaxis etc

• Means by which outbreak resolved

• Vaccination status pre/post outbreak

• Dose of vaccination as part of ORI

• Onward transmission from HCWs to patients

• Strategies for improving vaccination

• Study recommendations reported by authors

• Study limitations reported by authors

• Study limitations reported by the researcher

**Synthesis of results**

We will undertake a narrative synthesis of the evidence and conduct a summary of the descriptive statistics if feasible.

**References:**

1. WHO. Table 4: Summary of WHO Position Papers – Immunization of Health Care Workers: World Health Organisation; 2021 [Available from: <https://cdn.who.int/media/docs/default-source/immunization/immunization_schedules/immunization-routine-table4.pdf?sfvrsn=714e38d6_4&download=true>.

2. Immunize.org. Vaccine Timeline. Historic Dates and Events Related to Vaccines and Immunization. Immunize.org. 2021 [Available from: <https://www.immunize.org/timeline/>

3. Maltezou HC, Theodoridou K, Ledda C, Rapisarda V, Theodoridou M. Vaccination of healthcare workers: is mandatory vaccination needed? Expert Review of Vaccines. 2019;18(1):5-13.

4. Maltezou HC, Panagopoulos P, Sourri F, Giannouchos TV, Raftopoulos V, Gamaletsou MN, et al. COVID-19 vaccination significantly reduces morbidity and absenteeism among healthcare personnel: A prospective multicenter study. Vaccine. 2021;39(48):7021-7.

5. Imai C, Toizumi M, Hall L, Lambert S, Halton K, Merollini K. A systematic review and meta-analysis of the direct epidemiological and economic effects of seasonal influenza vaccination on healthcare workers. PLOS ONE. 2018;13(6):e0198685.

**Supplement 2:** Scoping Review: Ovid MEDLINE Search Strategy

Database: MEDLINE(R) All including Epub Ahead of Print, In-Process & Other Non-Indexed Citations, Daily and Versions(R) <1946-current>

Search Strategy:

--------------------------------------------------------------------------------

1 exp Disease Outbreaks/

2 outbreak$.tw.

3 cluster$.tw.

4 exp Epidemics/

5 epidemic$.tw.

6 1 or 2 or 3 or 4 or 5

7 exp Mycobacterium tuberculosis/

8 exp Tuberculosis/

9 (tuberculosis or tb).tw. s

10 exp Hepatitis B virus/

11 exp Hepatitis B/

12 "hepatitis b".tw.

13 exp Poliovirus/

14 exp Poliomyelitis/

15 polio$.tw.

16 exp Corynebacterium diphtheriae/

17 exp Diphtheria/

18 diphtheria$.tw.

19 exp Measles virus/

20 exp Measles/

21 measles.tw.

22 exp Rubella virus/

23 exp Rubella/

24 rubella$.tw.

25 exp Neisseria meningitidis/

26 exp Meningococcal Infections/

27 meningococc$.tw.

28 exp Influenza A virus/

29 exp Influenza B virus/

30 exp Influenza, Human/

31 (influenza or flu).tw.

32 exp Herpesvirus 3, Human/

33 exp Chickenpox/

34 (chickenpox$ or varicella$).tw.

35 exp Bordetella pertussis/

36 exp Whooping Cough/

37 (whooping adj1 cough$).tw.

38 pertussis.tw.

39 7 or 8 or 9 or 10 or 11 or 12 or 13 or 14 or 15 or 16 or 17 or 18 or 19 or 20 or 21 or 22 or 23 or 24 or 25 or 26 or 27 or 28 or 29 or 30 or 31 or 32 or 33 or 34 or 35 or 36 or 37 or 38

40 6 and 39

41 exp Health Personnel/

42 exp Laboratory Personnel/

43 exp Housekeeping, Hospital/

44 exp Ancillary Services, Hospital/

45 ((health$ or hospital$ or clinical$ or medical$ or nurs$ or lab$) adj3 (worker$ or employee$ or staff or personnel or provider$ or practitioner$ or profession$ or occupation$ or workforce or cleaner$ or orderl$ or ancillar$ or technician$)).tw.

46 (health adj1 care adj1 worker$).tw.

47 (hcw or doctor$ or nurse$ or allied health$).tw.

48 41 or 42 or 43 or 44 or 45 or 46 or 47

49 40 and 48

50 limit 49 to yr="2000 - 2022"

**Supplement 3: Preferred Reporting Items for Systematic reviews and Meta-Analyses extension for Scoping Reviews (PRISMA-ScR) Checklist**

| **SECTION** | **ITEM** | **PRISMA-ScR CHECKLIST ITEM** | **REPORTED ON PAGE #** |
| --- | --- | --- | --- |
| **TITLE** | | | |
| Title | 1 | Identify the report as a scoping review. | Click here to enter text. |
| **ABSTRACT** | | | |
| Structured summary | 2 | Provide a structured summary that includes (as applicable): background, objectives, eligibility criteria, sources of evidence, charting methods, results, and conclusions that relate to the review questions and objectives. | Click here to enter text. |
| **INTRODUCTION** | | | |
| Rationale | 3 | Describe the rationale for the review in the context of what is already known. Explain why the review questions/objectives lend themselves to a scoping review approach. | Click here to enter text. |
| Objectives | 4 | Provide an explicit statement of the questions and objectives being addressed with reference to their key elements (e.g., population or participants, concepts, and context) or other relevant key elements used to conceptualize the review questions and/or objectives. | Click here to enter text. |
| **METHODS** | | | |
| Protocol and registration | 5 | Indicate whether a review protocol exists; state if and where it can be accessed (e.g., a Web address); and if available, provide registration information, including the registration number. | Click here to enter text. |
| Eligibility criteria | 6 | Specify characteristics of the sources of evidence used as eligibility criteria (e.g., years considered, language, and publication status), and provide a rationale. | Click here to enter text. |
| Information sources* | 7 | Describe all information sources in the search (e.g., databases with dates of coverage and contact with authors to identify additional sources), as well as the date the most recent search was executed. | Click here to enter text. |
| Search | 8 | Present the full electronic search strategy for at least 1 database, including any limits used, such that it could be repeated. | Click here to enter text. |
| Selection of sources of evidence† | 9 | State the process for selecting sources of evidence (i.e., screening and eligibility) included in the scoping review. | Click here to enter text. |
| Data charting process‡ | 10 | Describe the methods of charting data from the included sources of evidence (e.g., calibrated forms or forms that have been tested by the team before their use, and whether data charting was done independently or in duplicate) and any processes for obtaining and confirming data from investigators. | Click here to enter text. |
| Data items | 11 | List and define all variables for which data were sought and any assumptions and simplifications made. | Click here to enter text. |
| Critical appraisal of individual sources of evidence§ | 12 | If done, provide a rationale for conducting a critical appraisal of included sources of evidence; describe the methods used and how this information was used in any data synthesis (if appropriate). | Click here to enter text. |
| Synthesis of results | 13 | Describe the methods of handling and summarizing the data that were charted. | Click here to enter text. |
| **RESULTS** | | | |
| Selection of sources of evidence | 14 | Give numbers of sources of evidence screened, assessed for eligibility, and included in the review, with reasons for exclusions at each stage, ideally using a flow diagram. | Click here to enter text. |
| Characteristics of sources of evidence | 15 | For each source of evidence, present characteristics for which data were charted and provide the citations. | Click here to enter text. |
| Critical appraisal within sources of evidence | 16 | If done, present data on critical appraisal of included sources of evidence (see item 12). | Click here to enter text. |
| Results of individual sources of evidence | 17 | For each included source of evidence, present the relevant data that were charted that relate to the review questions and objectives. | Click here to enter text. |
| Synthesis of results | 18 | Summarize and/or present the charting results as they relate to the review questions and objectives. | Click here to enter text. |
| **DISCUSSION** | | | |
| Summary of evidence | 19 | Summarize the main results (including an overview of concepts, themes, and types of evidence available), link to the review questions and objectives, and consider the relevance to key groups. | Click here to enter text. |
| Limitations | 20 | Discuss the limitations of the scoping review process. | Click here to enter text. |
| Conclusions | 21 | Provide a general interpretation of the results with respect to the review questions and objectives, as well as potential implications and/or next steps. | Click here to enter text. |
| **FUNDING** | | | |
| Funding | 22 | Describe sources of funding for the included sources of evidence, as well as sources of funding for the scoping review. Describe the role of the funders of the scoping review. | Click here to enter text. |

JBI = Joanna Briggs Institute; PRISMA-ScR = Preferred Reporting Items for Systematic reviews and Meta-Analyses extension for Scoping Reviews.

* Where *sources of evidence* (see second footnote) are compiled from, such as bibliographic databases, social media platforms, and Web sites.

† A more inclusive/heterogeneous term used to account for the different types of evidence or data sources (e.g., quantitative and/or qualitative research, expert opinion, and policy documents) that may be eligible in a scoping review as opposed to only studies. This is not to be confused with *information sources* (see first footnote).

‡ The frameworks by Arksey and O’Malley (6) and Levac and colleagues (7) and the JBI guidance (4, 5) refer to the process of data extraction in a scoping review as data charting*.*

§ The process of systematically examining research evidence to assess its validity, results, and relevance before using it to inform a decision. This term is used for items 12 and 19 instead of "risk of bias" (which is more applicable to systematic reviews of interventions) to include and acknowledge the various sources of evidence that may be used in a scoping review (e.g., quantitative and/or qualitative research, expert opinion, and policy document).

*From:* Tricco AC, Lillie E, Zarin W, O'Brien KK, Colquhoun H, Levac D, et al. PRISMA Extension for Scoping Reviews (PRISMAScR): Checklist and Explanation. Ann Intern Med. 2018;169:467–473. [doi: 10.7326/M18-0850](http://annals.org/aim/fullarticle/2700389/prisma-extension-scoping-reviews-prisma-scr-checklist-explanation).
