# Supplementary material for: Vaccine-Preventable Disease Outbreaks Among Healthcare Workers: A Scoping Review
Source: Clin Infect Dis. 2024 Apr 17;79(2):555–61. doi: 10.1093/cid/ciae209 (PMC11327795; doi:10.1093/cid/ciae209)
Supplement: ciae209_Supplementary_Data [file ciae209_supplementary_data.zip › Supplemental tables 2802clean.docx]

**Supplement 3: Studies describing outbreaks of influenza in healthcare workers since 2000**

| **Author, year** | **Country** | **Region** | **Income status** | **Year of outbreak** | **Duration** | **Setting** | **# ILI, attack rate** | **# HCW confirmed, attack rate** | **Occupation of HCW** | **Median age/Sex of HCW** | **Non HCW cases** | **Index case** | **Origin** | **Affected HCW Vaccinated^** | **Overall HCW vaccinated^** | **Transmission from HCW to patient** |
| --- | --- | --- | --- | --- | --- | --- | --- | --- | --- | --- | --- | --- | --- | --- | --- | --- |
| Alexander et al, 2010 (1) | Canada | AMR | High | 2009 | ~20 days | Long-term facility | 14 (patient confirmed) | NA, NA | NA | 33, NA | 9 residents | Residents | Nosocomial | NA | NA | NA |
| Apisarnthanarak et al, 2010 (2) | Thailand | SEAR | Upper Middle | 2009 | 3 days | Hospital | 4, 18% | 3, 14% | NA | Most F | NA | Patient | Nosocomial | NA | NA | No |
| Apisarnthanarak et al, 2008 (3) | Thailand | SEAR | Upper Middle | 2005- 2006 | 3 outbreaks (1-2 days) | Hospital | NA | 7,23%  4,18%  6,24% | NA | NA | NA | Patient | Nosocomial | NA | NA | Yes |
| Apisarnthanarak et al, 2006 (4) | Thailand | SEAR | Upper Middle | 2005 | 6 days | Hospital | 4, 13% | 3, 10% | NA | NA | NA | Patient | Nosocomial | NA | NA | NA |
| Aujayeb et al, 2013 (5) | UK | EUR | High | 2013 | 72-hour period | Hospital | 19, NA | 2, NA | NA | NA | 12 patients | NA | Nosocomial | NA | NA | NA |
| Badawi et al, 2016 (6) | Canada | AMR | High | 2014 | 13 days | Aged Care Setting | 2, NA (residents tested positive) | NA | NA | NA | 2 residents | Residents | Nosocomial | NA | 83% | NA |
| Balkhy et al, 2010 (7) | Saudi Arabia | EMR | High | 2009 | 6 months | Hospital | NA | 526, 5.4% | Doctors, nurses, others | Mean 34.5, 55.1% F | 1019 others | Various - patients, community | Nosocomial | NA | 40.2% | No |
| Banu et al 2011 (8) | India | SEAR | Lower Middle | 2009-2010 | 15 months | Hospital | 24, NA | 7, NA | 6 doctors, 1 nurse | NA, 6 doctors all M | 8 | NA | Community | 24/24 No | NA | NA |
| Bearden et al, 2012 (9) | USA | AMR | High | 2009 | 22 days | Hospital | 1, NA | 3, NA | NA | NA | 3 patients | Patient | Nosocomial | NA | NA | Yes |
| Benet et al, 2021 (10) | France | EUR | High | 2016 - 2017 | 7 months | Hospital | NA | 62, 22% | Doctors, nurses, others | 36, NA | NA | NA | Community-Acquired | NA | 50% | NA |
| Bhadelia et al, 2013 (11) | USA | AMR | High | 2009-2010 | 11 months | Hospital | 352, 3% | 141, 1.1% | Various | NA | NA | Various | Community-Acquired | NA | NA | NA |
| Bourlon et al, 2009 (12) | Mexico | AMR | Upper Middle | 2009 | 6 months | Hospital | 467, 16% | 96, 3% | NA | NA | 4 patients | NA | Community-Acquired | NA | NA | NA |
| Bradley-Stewart et al, 2014 (13) | Scotland | EUR | High | 2012 | 3 days | Laboratory | 6, 18% | 8/33, 24% | Laboratory staff | NA | NA | Lab worker | Community-Acquired | 93.9% vaccinated | NA | No |
| Bush et al, 2004 (14) | Australia | WPR | High | 2002 | 1 month | Aged Care Setting | 7, NA | 2, NA | Various | NA | 6 residents | 1 HCW, 1 resident | Nosocomial | 3/9 Yes, 6/9 No | 13 | Yes |
| Calatayud et al, 2009 (15) | France | EUR | High | 2008 | 24 days | Aged care facility | 4, 11%  (patient confirmed) | NA | Nurses | NA | 46 | HCW | Nosocomial | NA | 17% | Yes |
| Chan et al, 2012 (16) | Hong Kong | WPR | High | 2011 | 1 week | Hospital | NA | 1, 7% | NA | 35, 100% M | 7 patients | Patient | Nosocomial | NA | NA | No |
| Chang et al, 2007 (17) | Taiwan | WPR | High | 2004 | 3 weeks | Aged Care Setting | 8, 57% | NA | NA | 34, 100% F | 16 residents | HCW | Nosocomial | 8/8 Yes | 100% | Yes |
| Chen et al, 2011 (18) | USA | AMR | High | 2009 | 6 days | Hospital | 12, 6% (confirmed in patients) | NA | NA | NA | 4 patients | Patient | Nosocomial | NA | 57% | NA |
| Chen et al, 2010 (19) | Singapore | WPR | High | 2009 | 4 months | Hospital | NA | 22, NA | Mostly nurses | mean 35, 86% F | NA | NA | NA | 97% Yes | NA | NA |
| Cheng et al, 2010 (20) | China | WPR | Upper Middle | 2009 | 100 days | Hospital | NA | 12, 2% | NA | NA | 100 patients | NA | Nosocomial | NA | NA | No |
| Choi et al 2011 (21) | South Korea | WPR | High | 2010 | 2 months | Hospital | 739, 22% | 141, 4% | 17% doctors, 59% Nurses, 24% others | 76.9% <40 years, 74% F | NA | Various | Nosocomial | NA | 81% | NA |
| Chokephaibulkit et al, 2012 (22) | Thailand | SEAR | Upper Middle | 2009 | 3 months | Hospital | 120, 47% | 33, 13% | 81% nurses | 34, 93% F | NA | Community outbreak | Community acquired | 27/33 Yes, 5/33 No, 1/33 NA | 82% | NA |
| Chu et al 2013 (23) | Taiwan | WPR | High | 2009 - 2010 | 6 months | Hospital | NA | 51, 1% | 8 doctors, 31 nurses, 8 others, 4 non clinical | Mean 31.9, 84% F | NA | Community outbreak | Community acquired | 51/51 No | NA | NA |
| Considine et al, 2013 (24) | Australia | WPR | High | 2009 | NA | Health care facilities | 177, 36% | 12, 2.4% | 11 doctors, 1 nurse | NA | NA | Community outbreak | Community acquired | NA | NA | NA |
| Currie et al, 2011 (25) | Australia | WPR | High | 2009 | 1 month | Field Hospital | NA | 1, NA | 1 AH | NA | 47 patients | NA | Community-Acquired | NA | NA | NA |
| De Perio et al, 2012 (26) | USA | AMR | High | 2009 | 1 month | Hospital | 13, 15% | 5, 5.6% | 5 doctors | NA | NA | Various: nosocomial/community | Community-Acquired | NA | 90% | NA |
| Du et al. 2013 (27) | China | WPR | Upper Middle | 2009-2010 | 4 months | Hospital | NA | 171, 2% | 30 doctors, 102 nurses, 39 others | Mean 25.2, 72% F | 89 patients | 16 nosocomial, 48 community, rest community | Community-Acquired | NA | NA | Probable |
| Eibach et al, 2014 (28) | France | EUR | High | 2012 | 3 months | Hospital | NA | 6, 11% | NA | Mean 30.2, 100% F | 16 patients | Various: nosocomial/community | Community-Acquired | 2/6 Yes, 4/6 No | NA | NA |
| Fujita et al, 2020 (29) | Japan | WPR | High | 2019-2020 | 6 months | Hospital | NA | 16, 39% | NA | NA | 25 patients | 3 patients , 1 HCW | Nosocomial | 16/16 No | NA | NA |
| Fujita et al, 2008 (30) | Japan | WPR | High | 2005 | 3 months | Hospital | 13, NA | 17, NA | Doctors and nurses | NA | NA | Parties & nosocomial | Community-Acquired | Almost all vaccinated | NA | No |
| Gaillat et al 2008 (31) | France | EUR | High | 2005 | 10 days | Aged Care Setting | 6, 12.5% | NA | NA | NA | 32 residents (11 PCR +) | Resident | Nosocomial | NA | 41.7% | NA |
| Gallagher et al 2015 (32) | UK | EUR | High | 2012-2016 | 6 months (annual outbreaks) | Several aged care facilities | 207, NA | NA | NA | NA | >1000 | Various - community/nosocomial | Nosocomial | NA | 15% | NA |
| Laris Gonzales et al 2018 (33) | Mexico | AMR | Upper Middle | 2016 | 3 months | Pediatric hospital | 132, NA | 30, NA | Doctors, nurses, others | 36.5 years | NA | NA | Nosocomial | 48.7% Yes | NA | NA |
| Grund et al, 2010 (34) | Germany | EUR | High | 2008 | 22 days | Hospital | NA | 12, 12% | NA | NA | 5 patients | NA | Nosocomial | NA | ~40% | NA |
| Guy et al, 2005 (35) | Australia | WPR | High | 2001- 2002 | 1 month | Aged Care Setting | NA | 4, 21% | NA | 46, 66% | 19 patients | Visitor | Community-Acquired | 4/4 No | 7% | NA |
| Guy et al, 2004 (36) | Australia | WPR | High | 2002 | 2 outbreks: 18 days and 9 days | Aged Care Setting | NA | 7, 23%  13, 42%  Overall 20, 33% | NA | NA | 36 residents, 4 visitors | Patient | Nosocomial | NA | 8.3% | NA |
| Harriman et al 2009 (37) | USA | AMR | High | 2009 | 2 months | Nationwide | 8, NA | 18, NA | 12 nurses, 4 doctors, 10 others | NA, 83% F | NA | Community | Community acquired | 8/18 Yes, 10/18 No | NA | NA |
| Horcajada et al 2002 (38) | Spain | EUR | High | 2001 | 2 weeks | Hospital | 16, 28% | 13, 23% | 12 doctors, 13 nurses, 1 student, 3 others | NA | 8 patients | HCW - nurse aid | Nosocomial | 2/29 Yes, 27/29 No | 7% | NA |
| Huang & Cheng, 2017 (39) | Taiwan | WPR | High | 2016 | ~1 month | Hospital | NA | 1, 13% | NA | NA | 8 patients | Patient | Nosocomial | NA | NA | No |
| Ishikane et al, 2016 (40) | Japan | WPR | High | 2014- 2015 | 21 days | Aged Care Setting and hospital | NA | 36, 11% | Nurses and others | 40, 66% F | 62 residents/patients | NA | NA | 85.2% vaccinated | NA | NA |
| Jaeger et al, 2011 (41) | USA | AMR | High | 2009 | 27 days | Hospital and outpatient unit | NA | 9, 14% | Doctors, nurses, AH | 35, NA | 8 patients | Patients | Nosocomial | NA | 56% | NA |
| Rao et al, 2011 (42) | India | SEAR | Lower Middle | 2009- 2010 | 9 months | General Medical Clinic | NA | 2, NA | 2 doctors | NA | 18 patients | NA | Community-Acquired | NA | NA | NA |
| Javaid et al, 2021 (43) | USA | AMR | High | 2019 | 12 days | Hospital | 46, NA | 43, NA | Doctors, nurses and others | NA | 17 patients | Patient | Community-Acquired | 43/43 Yes | NA | Yes |
| Kay et al, 2011 (44) | USA | AMR | High | 2009 | ~1 week | Hospital | NA | 17, 53% | 17 doctors | 28.5 years, 94% F | NA | HCW | Nosocomial | NA | NA | NA |
| Khan et al, 2021 (45) | Bangladesh | SEAR | Lower Middle | 2007 | 10 days | Dormitory | 56, 47% | 5, 4% | Nursing students | 19.3, 100% F | NA | Teacher | Nosocomial | NA | NA | No |
| Kiertiburanakul et al, 2010 (46) | Thailand | SEAR | Upper Middle | 2009 | 3 months | Hospital | 370, NA | 81, NA | 19 doctors, 28 nurses, 8 medical students, 4 nursing students, 22 others | 75% between 20 and 29, 79% F | NA | Likely patient | Nosocomial | 21/81 Yes, 60/81 No | NA | NA |
| Kosnik, I. G et al, 2019 (47) | Slovenia | EUR | High | 2017 | 6 months | Hospital | 11, NA | 6, NA | NA | NA | 100 patients | Patient | Nosocomial | 9/17 Yes, 8/17 No | NA | No |
| Lobo et al, 2013 (48) | Brazil | AMR | Upper middle | 2009 | 4 months | Tertiary care teaching Hospital | NA | 52, NA | Doctors, physicians, others | 37.4, 60% F | NA | NA | Nosocomial | 27/52 Yes, 25/52 No | NA | NA |
| Magill et al, 2011 (49) | USA | AMR | High | 2009. | 24 days | Hospital | NA | 20, 26% | 6 doctors, 8 nurses, 6 others | Mean 34, 65% F | NA | Various | Nosocomial | 12/20 Yes, 8/20 No | NA | NA |
| Mahida et al, 2016 (50) | UK | EUR | High | NA | NA | Hospital | NA | 1, NA | NA | Range 44-97, NA | 16 patients | NA | Nosocomial | 1/1 No | NA | Yes |
| Malavaudet al, 2001 (51) | France | EUR | High | 2000 | 4 days | Hospital | NA | 3, 11% | 3 nurses | 36, 100% F | NA | Patient | Nosocomial | 3/3 No | NA | NA |
| Masse et al, 2016 (52) | France | EUR | High | 2015 | 3 months | Aged care facility | NA | 1, NA | NA | NA | 11 patients | NA | Nosocomial | NA | 15% | Likely |
| Matsushita et al, 2020 (53) | Japan | WPR | High | 2011-2017 | 5 different outbreaks | Hospital | NA | 39, NA | Doctors and nurses | NA | 35 patients | Various | Nosocomial | NA | NA | Yes |
| Mejia et al, 2012 (54) | Guatemala | AMR | Lower Middle | 2006 | 2 months | Hospital | NA | NA, 21% doctors, 10.5% others | Doctors and others | NA | 59 patients | NA | Nosocomial | NA | NA | NA |
| Miller et al, 2010 (55) | Scotland | EUR | High | 2009 | About 1 week | Hospital | NA | 7, NA | 6 doctors, 1 nurses | 29, 14% F | NA | Patient | Nosocomial | NA | NA | NA |
| Oguma et al, 2011 (56) | Japan | WPR | High | 2007 | 2 outbreaks, 2 weeks each | Hospital | NA | 13, NA | 2 doctors, 10 nurses, 1 other | mean 41.1, NA | 11 patients | Patient | Community-Acquired | 9/13 Yes, 4/13 No | 80% | NA |
| Ohara et al, 2011 (57) | Japan | WPR | High | 2009 | 10 days | Dialysis center | NA | 11, 22% | Doctors, nurses, others | 33, 64% | NA | NA | Nosocomial | 11/11 Yes | NA | NA |
| O’Meara et al, 2006 (58) | Ireland | EUR | High | 2005 | ~1 month | Hospital | 17, 18% (confirmed in patient) | NA | NA | NA | 37 patients | NA | Nosocomial | 3/17 Yes, 14/17 No | NA | NA |
| Perez-Padilla et al, 2009 (59) | Mexico | AMR | Upper Middle | 2009 | 1 month | Hospital | 19, NA | 3, NA | 3 nurses | NA | 18 patients | Patients | Nosocomial | NA | NA | Yes |
| Pryluka et al, 2013 (60) | Argentina | AMR | Upper Middle | 2009 | 4 months | 51 hospitals | 41, <1% | 19, <1% | NA | NA | NA | NA | Community-Acquired | NA | NA | NA |
| Raymond et al, 2012 (61) | New Zealand | WPR | High | 2009 | 3 months | Hospital | NA | 103, 2% | NA | NA | NA | NA | Nosocomial | NA | NA | NA |
| Risa et al, 2009 (62) | USA | AMR | High | 2006 | 5 days | Behaviour Health unit | 8, 20% | NA | Nurses, physicians, support staff | NA | 6 patients | Patient | Nosocomial | NA | 55% | NA |
| Sayers et al, 2012 (63) | Ireland | EUR | High | 2008 | 2 weeks | Hospital | 6, 9% | 2, 3% | NA | 40, NA | 21 patients | Patient | Community-Acquired | NA | 3% | NA |
| Schulz-Stubner et al, 2019 (64) | Germany | EUR | High | 2013-2018 | Several | Various | NA | 22, NA | NA | NA | NA | NA | NA | NA | NA | NA |
| Sunagawa et al, 2015 (65) | Japan | WPR | High | 2011 | One week | Hospital | NA | 8, NA | NA | NA | 10 patients | HCW | Nosocomial | NA | 100% | Yes |
| Tennant et al, 2020 (66) | Australia | WPR | High | 2017 | 123 influenza outbreaks across 106 facilities | Aged Care Setting | NA | NA, 0.3% | NA | NA | NA | NA | Nosocomial | NA | 39% | NA |
| Tsagris et al, 2012 (67) | Greece | EUR | High | 2011 | 2 weeks | Hospital | 5, 9% | 3, 6% | 3 nurses | NA | 3 neonates | HCW | Nosocomial | 8/8 No | 15% | NA |
| Ursic et al, 2016 (68) | Slovenia | EUR | High | 2011-2012 | 11 days | Aged Care Setting | NA | 9, NA | NA | NA | 56 residents | NA | Nosocomial | NA | 20% | NA |
| Vilar-Compte et al, 2011 (69) | Mexico | AMR | Upper Middle | 2009 | 21 days | Hospital | 24, NA | 7, NA | 7 doctors | 30.7, NA | NA | Patients | Nosocomial | NA | NA | NA |
| Vilella et al, 2012 (70) | Dominican Republic | AMR | Upper Middle | 2009 | 2 weeks | Travelling medical students | NA | 37, 33% | 37 students | 24, 67% F | NA | NA | Community-Acquired | NA | 6% | NA |
| WHO, 2014 (71) | Egypt | EMR | Lower Middle | 2013-2014 | 2 months | Hospital | NA | 5, NA | NA | NA | 70 | NA | Nosocomial | NA | NA | NA |
| Wilson et al, 2019 (72) | USA | AMR | High | 2017 | 2 weeks | Hospital | 34, NA | 16, NA | 3 doctors, 8 nurses, 5 others | NA | 10 patients | Likely a patient | Nosocomial | 15/16 Yes, 1/16 No | NA | NA |
| Win et al, 2010 (73) | Singapore | WPR | High | 2007 | 1 week | Long term facility | NA | 2, 6.7% | 2 nurses | 24, NA | 17 residents | NA | Nosocomial | 2/2 Yes | 100% | NA |
| Wise et al, 2011 (74) | USA | AMR | High | 2009 | 2 months | Various | NA | 70, NA | Doctors, nurses and others | Mean 38, 76% F | NA | Various (35 nosocomial) | Nosocomial | 62% vaccinated | NA | NA |
| Wong et al, 2011 (75) | Hong Kong | WPR | Upper Middle | 2009 | Several days | Hospital | NA | 3, NA | 1 doctor, 1 nurse, 1 other | NA | 2 patients | Patient | Nosocomial | NA | NA | NA |
| Zhang et al, 2013 (76) | China | WPR | Upper Middle | 2009-2010 | 5 months | Hospital | NA | 51, NA | 21 doctors, 24 nurses, 6 others | 70% 18-29, 82% F | NA | Various | Nosocomial | 4/51 Yes  47/52 No | 14% | NA |

^prior to outbreak

Nurses include nursing assistants and midwives

AH allied health, AMR Region of the Americas, EMR Eastern Mediterranean region, EUR European region, F female, HCW health care worker, ILI influenza like illness, NA not applicable, SEAR South East Asia region, USA United States of America, UK United Kingdom, WPRO Western Pacific region

**Supplement 4: Studies describing outbreaks of measles in healthcare workers since 2000**

| **Author, year** | **Country** | **Region** | **Income status** | **Year of outbreak** | **Duration** | **Setting** | **# HCW, attack rate** | **Occupation of HCW** | **Median age/Sex of HCW** | **Non HCW cases** | **Index case** | **Origin** | **Affected HCW Vaccinated^** | **Overall HCW vaccinated^** | **Transmission from HCW to patient** |
| --- | --- | --- | --- | --- | --- | --- | --- | --- | --- | --- | --- | --- | --- | --- | --- |
| Agut-Basquet et al, 2016 (77) | Spain | EUR | High | NA | NA | Hospital | 1, NA | NA | range 1-31, NA | 5 patients | Community cluster (131 cases) | Community-Acquired | 1/1 Yes | 6% had low level immunity | NA |
| Augusto et al, 2019 (78) | Portugal | EUR | High | 2017 | 6 months | Hospital | 12, NA | NA | NA | 15 others | Various patients | Nosocomial | 7/12 Yes, 2/12 No, 3/12 NA | NA | No |
| Augusto et al, 2018 (79) | Portugal | EUR | High | 2018 | 10 weeks | Hospital | 89, NA | 34 doctors, 20 nurses, 16 AH, 18 medical/nursing students, 1 other | mean 30, 58% F | 23 others | Various patients | Nosocomial | 7/89 Yes, 76/89 partially, 6/89 No | NA | Yes |
| Barbadoro et al, 2013 (80) | Italy | EUR | High | 2011 | 3 months | Hospital | 4, 5.7% | 3 nurses, 1 student | NA, 50% F | NA | Child | Nosocomial | 1/4 Yes, 2/4 No, 1/4 NA | 80% | No |
| Baxi et al, 2013 (81) | UK | EUR | High | NA | 1 month | Hospital | 1, NA | NA | NA | 3 patients | Patient | Nosocomial | 1/1 Yes | NA | Yes |
| Beard et al, 2011 (82) | Australia | WPR | High | 2010 | 2 months | Hospital | 2, NA | NA | 37, 100% F | 7 patients | Returned traveler | Community-Acquired | 2/2 No | NA | Yes |
| Berry et al, 2019 (83) | England | EUR | High | 2017 | 16 days | Hospital | 6, 4.5% | 1 paramedic, 5 NA | NA | 2 others | Community member | Nosocomial | 4/6 Yes, 1/6 No, 1/6 NA | NA | No |
| Biron et al, 2010 (84) | France | EUR | High | 2008-2009 | 13 months | Hospital | 4, NA | 2 nurses, 1 AH, 1 medical student | NA | 9 others | NA | NA | 2/4 No, 2/4 NA | NA | NA |
| Bogowicz et al, 2014 (85) | England | EUR | High | NA | NA | Hospital | 2, NA | 2 doctors | 30.5, 0% F | NA | Patient | Nosocomial | 2/2 Yes | 90% | No |
| Botelho-Nevers et al, 2013  (86) | France | EUR | High | 2010 | 11 months | Hospital | 14, 0.1% | 7 doctors, 4 nurses, 3 medical students | mean 27.5, 64% F | NA | Various (2 likely fom community) | Nosocomial | 4/14 partially, 6/14 No, 2/14 NA | 93% | No |
| Burgess et al, 2013 (87) | Australia | WPR | High | 2011 | 32 days | GP surgery, hospital | 1, NA | 1 nurse | 36, 100% F | 3 others | HCW | NA | 1/1 partially | NA | Yes |
| Chen et al, 2011 (88) | USA | AMR | High | 2008 | 4 months | Hospital | 1, NA | NA | 41, 100% F | 13 others | Returned traveler | Community-Acquired | NA | 98% | No |
| Cheng et al, 2019 (89) | Hong Kong | WPR | High | 2019 | 2 months | Hospital | 2, 3% | 1 nurse, 1 other | 21.5, 50% F | 33 others | Airport worker | Nosocomial | 2/2 Yes | 96% | No |
| Choi et al, 2011 (90) | South Korea | WPR | High | 2007 | 21 weeks | Hospital | 3, NA | 2 nurses 1 doctor | NA | 452 others | various patients | Community-Acquired | NA | NA | Yes |
| Corbin et al, 2013 (91) | France | EUR | High | 2009-2011 | 20 months | Hospital | 19, NA | 5 doctors, 7 nurses, 2 nursing students, 5 others | NA | NA | 14 community, 5 nosocomial | Community-Acquired | 15/19 Yes, 4/15 partially | NA | NA |
| Cornelissan et al, 2020 (92) | Belgium | EUR | High | 2016-2017 | 6 months | Hospital | 36, NA | NA | 30, NA | 253 others | 17 Nosocomial, 2 community, 2 NA | Community | 13/36  partially, 7/36 No, 6/36 NA | NA | Yes |
| Currie et al, 2017 (93) | Wales | EUR | High | 2017 | 4 months | General Medical Clinic | 1, NA | NA | NA | NA | Patient | Community | NA | NA | NA |
| Davidson et al, 2002 (94) | Australia | WPR | High | 2001 | 2 months | Community outbreak | 1, NA | NA | 29, 100% F | 49 others | Community member | Community | 1/1 No | NA | NA |
| DeSwart et al, 2000 (95) | Netherlands | EUR | High | 2017 | NA | Hospital | 3, NA | NA | 24, NA | NA | Paediatric patient | Nosocomial | 3/3 No | NA | No |
| Dina et al, 2017 (96) | France | EUR | High | 2016 | NA | Hospital | 3, NA | 1 nurse, 2 unknown | NA | 10 others | Refugee, 1 acquired in camp, 2 nosocomial | Community-Acquired | 1/3 No, 2/3 NA | NA | No |
| Filia et al, 2017 (97) | Italy | EUR | High | 2017 | 8 months, ongoing at time of publication | Community outbreak | 296, NA | NA | 33, NA | 4181 others | NA | Community-Acquired | 36/296  partially, 239/296 No, 6/296 NA | NA | NA |
| Filia et al, 2015 (98) | Italy | EUR | High | 2014 | 6 months | Community outbreak and hospital | 15, NA | NA | NA | 65 others | Cruise ship passenger | Community-Acquired | NA | NA | NA |
| Fu et al, 2018 (99) | China | WPR | Upper Middle | 2018 | 2 weeks | Hospital | 11, 0.4% | 2 students, 9 NA | 26, 64% F | NA | NA | Nosocomial | 1/11 Yes, 2/11 No, 8/11 NA | NA | No |
| Garcia Comas et al, 2017 (100) | Spain | EUR | High | 2011-2012 | 77 weeks | Community outbreak | 24, NA | NA | NA | 765 | 13 Nosocomial, 5 community | Community-Acquired | NA | NA | Yes |
| Garcia Comas et al, 2010 (101) | Spain | EUR | High | 2006 | 6 months | Hospital | 18, NA | NA | NA | NA | Various | Community-Acquired | NA | NA | NA |
| Georgakopoulou et al, 2018 (102) | Greece | EUR | High | 2017-2018 | 12 months | Nationwide | 94, NA | NA | NA | 2565 others | NA | Community-Acquired | All partially or not vaccinated | NA | NA |
| Georgakopoulou et al, 2018 (103) | Greece | EUR | High | 2017 | 13 months | Community outbreak and hospital | 129, NA | 47 doctors, 43 nurses, 11 paramedics, 28 others | 39, NA | 3021 others | NA | Community-Acquired | 39/129 partially, 48/129 No, 43/129 NA | NA | NA |
| George et al, 2017 (104) | Portugal | EUR | High | 2017 | 4 months | Community outbreak and Hospital | 13, NA | 5 doctors, 5 nurses, 3 others | NA | 15 others | Community case | Community-Acquired | 10/13 Yes, 3/13 No | NA, | NA |
| Gohil et al, 2016 (105) | USA | AMR | High | 2014 | 3 months | Community outbreak and Hospital | 5, NA | NA | Range, 32-41, 60% F | 17 others | Community case | Community-Acquired | 4/5 Yes, 1/5 No | NA | No |
| Grammens et al, 2017 (106) | Belgium | EUR | High | 2016- 2017 | 3 months, ongoing | Community outbreak | 18, NA | NA | NA | 159 others | Community case | Community-Acquired | NA | NA | NA |
| Grammens et al, 2016 (107) | Belgium | EUR | High | 2016 | 6 months | Community outbreak and hospital | 4, NA | NA | NA | 63 others | Community case | Community-Acquired | 3/4 No, 1/4 NA | NA | NA |
| Green et al, 2012 (108) | USA | AMR | High | 2009 | NA | Hospital | 1, NA | 1 Doctor | NA | 5 others | Returned traveler | Community-Acquired | 1/1 Yes | NA | No |
| Grgic-Vitek et al, 2010 (109) | Slovenia | EUR | High | 2010 | 2 weeks | Hospital | 1, NA | 1 HCW | 39, 100% F | 3 patients | Patient | Nosocomial | 1/1 Yes | NA | No |
| Guaita Calatrava et al, 2011 (110) | Spain | EUR | High | 2011 | 8 months, ongoing | Hospital | 13, NA | 7 doctors, 5 nurses, 1 other | 30 years | 8 | Roma traveler | Community-Acquired | 69.2% No | NA | Yes |
| Hahne et al, 2016 (111) | Netherlands | EUR | High | 2014 | 3 weeks | Hospital | 8, 8% | NA | 27, 50% F | NA | 2 patients | Nosocomial | 6/8 Yes, 1/8 partially, 1/8 NA | 70% partially vaccinated | Yes |
| Hebert et al, 2017 (112) | Belgium | EUR | High | 2017 | 2 months | Hospital | 8, NA | NA | NA | 35 others | Returned traveler | Community-Acquired | NA | 88% | NA |
| Hiller et al, 2019 (113) | Germany | EUR | High | 2017 | 2 months | Hospital | 10, NA | 2 doctors, 6 nurses, 2 others | 33.5, 60% F | NA | Patient | Nosocomial | 1/10 Yes, 1/10 partially, 8/10 No | NA | Yes |
| Huoi et al, 2012 (114) | France | EUR | High | 2010-2011 | 17 months | Hospital | 16, NA | NA | NA | 391 | NA | Community acquired | NA | NA | NA |
| Jane et al, 2015 (115) | Spain | EUR | High | 2006 – 2014 | 9 years (4 separate outbreaks) | Community outbreak | 3% of 381 in 2006, 6% of 289 in 2011, 5.4% of 54 in 2013, 23.4% of 124 in 2014 | NA | NA | NA | Patient | Community acquired | NA | NA | NA |
| Jia et al, 2018 (116) | China | WPR | Upper Middle | 2016 | 2 months | Hospital | 19, 2% | Nurse, doctors and others | 26, 53% F | NA | HCW - Nurse | Nosocomial | 2/19 partially, 1/19 No, 16/19 NA | 68% vaccinated | No |
| Jones et al, 2016 (117) | France | EUR | High | 2016 | 2 months | Refugee camp | 3, NA | NA | NA | 10 others | Refugee | Community-Acquired | 2/3 Yes, 1/3 No | NA | NA |
| Jones et al, 2015 (118) | USA | AMR | High | 2015 | ~ 1 month | Outpatient facility | 1, 1% | NA | NA, 100% F | 0 | Patient | Community-Acquired | 1/1 Yes | 100% vaccinated | No |
| Kohnen et al, 2021 (119) | Luxembourg | EUR | High | 2019 | NA | Hospital | 3, NA | NA | NA | 18 others | HCW - unvaccinated student | Community-Acquired | 3/3 Yes | NA | No |
| Komitova et al, 2011 (120) | Bulgaria | EUR | Upper Middle | 2009-2010 | 7 months | Hospitals and primary care | 40, NA | 19 doctors, 7 nurses, 14 others | 38, 74% F | 286 others | Patients | Nosocomial | 1/40 Yes, 17/40 NA | NA | NA |
| Laure et al, 2020 (121) | France | EUR | High | 2017-2018 | 7 months | Hospital | 28, NA | NA | NA | 143 patients | NA | Community acquired | Most partially  vaccinated | NA | NA |
| Lee et al, 2022 (122) | Taiwan | WPR | High | 2008 | 2 months | Hospital | 1, NA | NA | 39 | 7 children | Patient | Nosocomial | NA | NA | NA |
| Maltezou et al, 2018 (123) | Greece | EUR | High | 2017-2018 | 10 months | Various | 117, NA | 45 doctors, 34 nurses, 38 others | NA | NA | Community wide | Community acquired | 6/117 Yes, 34/117 partially, 59/117 No  8/117 NA | NA | NA |
| Medić et al, 2019 (124) | Serbia | EUR | Upper Middle | 2014-2015 | About 1 year | Community outbreak and healthcare facilities | 42, NA | 12 doctors, 20 nurses, 2 medical students, 8 others | NA | 378 | HCW - University student | Community-acquired | 8/42 Yes, 6/42 No, 28/42 NA | NA | Yes |
| Moghadami et al, 2014 (125) | Iran | EMR | Lower Middle | 2012 | 5 weeks | Community outbreak | 1, NA | 1 rural health worker | 35, 100% F | 5 others | Patient | Community acquired | 1/1 Yes | NA | No |
| Monsel et al, 2011 (126) | France | EUR | High | 2007-2009 | 28 months | 4 Hospitals and community | 6, NA | 5 doctors, 1 nurse | NA | 15 patients | NA | NA | 2/6 Yes, 3/6 partially, 1/6 No | NA | NA |
| Nascimento et al, 2020 (127) | Brazil | AMR | Upper Middle | 2020 | NA | Hospital | 1, NA | 1 doctor | 25, 0% F | 0 | Unknown | NA | 1/1 Yes | NA | NA |
| Orsi et al, 2019 (128) | Italy | EUR | High | 2017-2018 | 3 months | Hospital | 5, NA | 2 doctors, 2 nurses, 1 AH | NA | 29 others | Patient | Community-Acquired | 5/5 No | NA | Yes |
| Porretta et al, 2017 (129) | Italy | EUR | High | 2017 | 3 months | Hospital | 15, NA | NA | NA | 19 others | HCW | Nosocomial | 2/15 Yes, 11/15 No, 2/15 NA | NA | Yes |
| Ragusa et al, 2020 (130) | Italy | EUR | High | 2017-2018 | 13 months | Community outbreak | 14, NA | NA | NA | 829 others | Not reported | Community-acquired | 14/14 No | NA | NA |
| Rana et al, 2020 (131) | UK | EUR | High | 2016 | 4 months | Community outbreak | 11, NA | NA | NA | 171 others | Community member | Community-acquired | 2/11 No, 9/11 NA | NA | Yes |
| Rota et al, 2011 (132) | USA | AMR | High | 2009 | NA | Hospital | 2, NA | 2 doctors | 38, NA | 6 others | Patient | Community-Acquired | 2/2 Yes | NA | No |
| Sá Machado et al, 2018 (133) | Portugal | EUR | High | 2018 | 7 weeks | Hospital | 173, 4% | 49 doctors, 60 nurses, 24 medical/nursing students, 40 others | range 1-59, 64% F | 38 others | NA | Nosocomial | 70% vaccinated | NA | Yes |
| Sillam et al, 2009 (134) | France | EUR | High | 2008 | 2 months | Hospital | 5, 4% | NA | NA | 31 patients | Returned traveler | Nosocomial | 1/5 Yes, 4/5 NA | 100% | No |
| Six et al, 2010 (135) | France | EUR | High | 2018 | 11 months | Hospital and community outbreak | 28, NA | 4 nurses, 4 doctors, 11 students, 9 others | NA | 356 others | NA | Community | 8/28 partially, 14/28 No, 6/28 NA | NA | NA |
| Song et al, 2022 (136) | South Korea | WPR | High | 2019 | 2 months | Hospital | 22, NA | NA | NA, 77% F | 4 patients | NA | Nosocomial | 12/22 Yes, 3/22 partially, 1/22 No, 6/22 NA | Most vaccinated | NA |
| Tafuri et al, 2009 (137) | Italy | EUR | High | 2008 | NA | Hospital | 1, NA | 1 nurse | 39, 100% F | NA | Patient | Nosocomial | 1/1 No | NA | No |
| Tajima et al, 2014 (138) | Japan | WPR | High | 2003 | 2 weeks | Hospital | 1, NA | 1 Nurse | 31, 100% F | NA | Patient | Nosocomial | NA | 94% immune by serology | No |
| Terada et al, 2001 (139) | Japan | WPR | High | 2000 | ~2 months | Hospital | 15, NA | Doctors, nurses, medical students | 20s-30s, NA | NA | NA | Nosocomial | NA | NA | No |
| Torner et al, 2021 (140) | Spain | EUR | High | 2018 | 4 months | Hospital | 8, NA | 5 clinical, 3 non-clinical | NA | 6 patients | Patient | Nosocomial | 3/8 Yes, 5/8 NA | NA | No |
| Torner et al, 2015 (141) | Spain | EUR | High | 2001-2013 | Multiple outbreaks (26) | Community outbreak | 52, NA | NA | NA | 745 others | Various | Nosocomial | 2/52 Yes, 4/56 partially, 46/52 No | NA | NA |
| Trmal et al, 2015 (142) | Czech Republic | EUR | High | 2014 | 7 months | Hospital and community outbreak | 14, NA | 2 Doctors, 9 nurses, 3 others | NA | 291 | Returned traveler | Community-Acquired | NA | NA | Yes |
| Trmal et al, 2014 (143) | Czech Republic | EUR | High | 2014 | 2 months | Hospital and community outbreak | 68, NA | Doctors, nurses and other health care workers | NA | 103 others | Returned traveler | Community-Acquired | NA | NA | Yes |
| Unknown, 2008 (144) | USA | AMR | High | 2008 | 1 month | Hospital | 1, NA | nurse | NA | 10 others | Returned traveler | Community-acquired | 1/1 No | NA | NA |
| Unknown, 2018 (145) | USA | AMR | High | NA | NA | Hospital | 1, NA | nurse | NA | 75 others | Child in ED | Community-Acquired | 1/1 Yes | NA | No |
| Vainio et al, 2012 (146) | Norway | EUR | High | 2011 | Four outbreaks | Hospital | 4, NA | NA | NA | 29 others | Various- overseas and nosocomial | Community-Acquired | 2/4 Yes, 1/4 No, 1/4 NA | NA | NA |
| Vink et al, 2020 (147) | England | EUR | High | 2018 | 8 weeks | Hospital | 9, 4% | NA | NA | 25 others | Patient in ICU | Nosocomial | 2/9 Yes, 7/9 NA | 91% | NA |
| Westgeest et al, 2020 (148) | Europe | EUR | High | 2018 | 2 separate cases | Hospital | 2, NA | 1 doctor, 1 nurse | 45.5, 100% F | NA | Patient | Nosocomial | 2/2 No | NA | NA |
| Weston et al, 2006 (149) | Australia | WPR | High | 2003 | 1 month | Community outbreak | 2, NA | NA | 34, NA | 7 others | Returned traveler | Community-Acquired | 1/2 maybe, 1/2 No | NA | No |
| Zhang et al, 2016 (150) | China | WPR | Upper Middle | 2015 | 20 days | Hospital | 50, NA | 7 doctors, 31 nurses, 12 others | NA | 10 patients | Patient | Nosocomial | NA | 73% | NA |
| Zmerli et al, 2021 (151) | Lebanon | EMR | Lower Middle | 2018 | 4 months | Hospital | 9, 9% | Doctors, nurses, medical students | Range 23-33 | 8 patients | Patient | Nosocomial | 9/9 Yes | NA | NA |

^prior to outbreak

Nurses include nursing assistants and midwives

AH allied health, AMR Region of the Americas, EMR Eastern Mediterranean region, ED emergency unity, EUR European region, F female, HCW health care worker, ICU intensive care unit, NA not applicable, SEAR South East Asia region, USA United States of America, UK United Kingdom, WPRO Western Pacific region

**Supplement 5: Studies describing outbreaks of varicella in healthcare workers since 2000**

| **Author, year** | **Country** | **Region** | **Income group** | **Year of outbreak** | **Duration** | **Setting** | **# HCW, attack rate** | **Occupation of HCW** | **Median age/Sex of HCW** | **Non HCW cases** | **Index case** | **Origin** | **Affected HCW Vaccinated^** | **Transmission from HCW to patient** |
| --- | --- | --- | --- | --- | --- | --- | --- | --- | --- | --- | --- | --- | --- | --- |
| Alanazi et al, 2020 (152) | Saudi Arabia | EMR | High | 2018 | 17 days | Hospital | 3, NA | 1 doctor, 2 nurses | NA | 6 patients | Patient | Nosocomial | NA | No |
| Aly et al, 2007 (153) | Kuwait | SEAR | Lower Middle | NA | ~21 days | Hospital | 3, NA | 3 nurses | 26, 100% F | NA | Patient | Nosocomial | 2/3 No, 1/3 NA | No |
| Apisarnthanarak et al, 2007 (154) | Thailand | SEAR | Upper Middle | 2004 | 33 Days | Hospital | 10 (7%) | NA | 21, 100% F | NA | Patient | Nosocomial | NA | No |
| Behrman et al, 2003 (155) | United States | AMR | High | 2000 | NA | Hospital | 5, NA | 3 nurses, 2 AH | 27, 60% F | NA | NA | NA | NA | NA |
| Bhatti et al, 2014 (156) | India | SEAR | Lower Middle | 2012 | 20 days | Hospital (HCW accomodation) | 8, NA | 8 nursing students | 20, 100% F | No | HCW | NA | 2/8 Yes, 6/8 NA | No |
| Gunawan et al, 2010 (157) | Indonesia | SEAR | Lower Middle | 2009 | 39 days | Hospital | 1, NA | 1 non clinical | NA | 4 children, 1 family member | Patient | Nosocomial | No | NA |
| Leung et al, 2010 (158) | United States | AMR | High | 2009 | 34 days | Long term facility | 2, NA | 1 nurse, 1 other clinical | 30.5, NA | 11 residents | ?Close contact of HCW | Community | 2/2 No | No |
| Lopez et al, 2008 (159) | United States | AMR | High | 2004 | 20 days | Long term facility | 1, NA | NA | 29 | 2 residents | Patient | Nosocomial | NA | NA |
| Park et al, 2013 (160) | Korea | WPR | High | 2009 | 16 days | Hospital (rehab centre) | 1, NA | NA | NA | 15 others | Visitor | Nosocomial | NA | NA |
| Paul & Jacob, 2006 (161) | India | SEAR | Lower Middle | NA | 18 days | Hospital | 4 (22%) | 1 doctor, 3 other clinical | 22.5 (mean) | No | Patient (deseased) | Nosocomial | NA | No |
| Saidel-Odes et al, 2010 (162) | Israel | EUR | High | 2007 | 21 days | Hospital | 3, NA | 3 nurses | 29, 66% F | NA | Patient | Nosocomial | 3/3 No | No |
| Sarit et al, 2015 (163) | India | SEAR | Lower Middle | 2015 | NA | Hospital | 8, NA | 1 doctor, 7 nurses | NA | No | Patient | Nosocomial | 1/8 No, 7/8 NA | NA |
| Sharma et al, 2021 (164) | India | SEAR | Lower Middle | NA | 38 days | Hospital | 5, NA | 2 doctors, 3 nurses | NA | No | Patient | Nosocomial | NA | No |
| Singh et al, 2022 (165) | India | SEAR | Lower Middle | 2017 | 21 days | Hospital | 6, NA | 2 Nurses, 4 other clinical | 30 (mean), 25% F | No | HCW (exposed to patient elsewhere) | Nosocomial | 1/6 Yes, 5/6 NA | NA |
| Sood, 2013 (166) | India | SEAR | Lower Middle | NA | ~2 months | Hospital | 14, NA | 10 nurses, 1 doctor, 3 other clinical | NA | No | Patient | Nosocomial | 14/14 No | No |
| Yang et al, 2019 (167) | China | WPR | Upper Middle | Multiple outbreaks: 2013 -2017 | NA | Hospital | 4, NA | 1 doctor, 2 nursing students, 1 other non-clinical | NA | 4 others | NA | NA | 3/4 No, 1/4 NA | No |

^prior to outbreak

Nurses include nursing assistants and midwives

AH allied health, AMR Region of the Americas, EMR Eastern Mediterranean region, EUR European region, F female, HCW health care worker, NA not applicable, SEAR South East Asia region, USA United States of America, UK United Kingdom, WPRO Western Pacific region

**Supplement 6: Studies describing outbreaks of tuberculosis in healthcare workers since 2000**

| **Author, year** | **Country** | **Region** | **Income status** | **Year of outbreak** | **Duration** | **Setting** | **# HCW, attack rate (TB disease)** | **# HCW, attack rate (TB infection)** | **Occupation of HCW** | **Median age/Sex of HCW** | **Non HCW cases** | **Index case** | **Origin** | **Transmission from HCW to patient** |
| --- | --- | --- | --- | --- | --- | --- | --- | --- | --- | --- | --- | --- | --- | --- |
| Borgia et al, 2011 (168) | Italy | EUR | High | 2011 | 3 years | Hospital | 1, NA | NA | 1 nurse | NA | 1 neonate, 118/1340 neonates IGRA positive | HCW- nurse | Nosocomial | Yes |
| Chen et al, 2010 (169) | Taiwan | WPR | High | 2006 | 6 months | Hospital | 15, 43% | NA | 11 nurses, 4 doctors | NA | NA | Likely infected patient | Nosocomial | No |
| Diel et al, 2005 (170) | Germany | EUR | High | 1997-2002 | 5 years | Hospital | 8, NA | NA | 4 nurses, 2 doctors, 2 other clinical | range 21-55, NA | NA | Various patients (each HCW linked to separate case) | Nosocomial | NA |
| Harris et al, 2013 (171) | USA | AMR | High | 2007 | NA | Hospital and Long-term facility | 0, NA | 5, 2% (TST) | NA | NA | 7 TST conversion in patients (11%) | Patient | Nosocomial | No |
| Hazard et al, 2016 (172) | USA | AMR | High | 2010-2014 | 4 years | Hospital (food services) | 4, NA | 20, 9% (TST) | 4 food service employees | NA | 0 | HCW- food service employee | Nosocomial | NA |
| Holden et al, 2018 (173) | UK | EUR | High | NA | NA | Hospital | 1, NA | 8, 19% (IGRA)* | NA | NA | NA | Patient | Nosocomial | No |
| Huang et al, 2007 (174) | Taiwan | WPR | High | 2003-2004 | 12 months | Hospital | 66, 4% | NA | 35 nurses, 3 doctors, 2 AH, 12 other clinical, 14 others | NA | 13 patients | 1 patient on a respirator | Nosocomial | NA |
| Jonsson et al, 2013 (175) | Sweden | EUR | High | 2008 | 6 months | Hospital | 3, NA | 15, 42% (TST)* | NA | NA | 4 active, 4 TST positive patients | Patient | Nosocomial | NA |
| Kazama et al, 2013 (176) | Japan | WPR | High | 2010 | 12 months | Hospital | 3, 6% | 13, 22% | NA | NA | NA | Patient | Nosocomial | No |
| Khalil et al, 2013 (177) | Canada | AMR | High | 2010 | 8 months | Aged Care Setting | 1, 0.8% | 9, 7.4% (TST) | NA | NA | 3 residents (all died) active TB, 15 TST positive | HCW - Staff member | Unknown | Yes |
| Lai et al, 2016 (178) | Taiwan | WPR | High | 2011 | 13 months | Aged Care Setting | 1, 5.5% | NA | NA | NA | NA | Patient | Nosocomial | No |
| Laniado-Labaro & Navarro-Alvarez, 2007 (179) | Mexico | AMR | Upper Middle | 2005 - 2006 | 7 months | Hospital | 17, NA | 78, NA (TST)* | 13 doctors, 2 nurses, 2 janitors | 25, 59% F (active) | NA | Unknown | Nosocomial | NA |
| Luzzati et al, 2017 (180) | Italy | EUR | High | NA | 12 months | General Medical Clinic | 1, NA | 6, NA (TST/IGRA)* | NA | NA | 6 patients | Patient | Nosocomial | NA |
| Malone et al, 2004 (181) | USA | AMR | High | 2001-2002 | 12 months | 3 Hospitals | 0, NA | 5, NA (TST)* | NA | NA | NA | Patient | Nosocomial | NA |
| McLaughlin et al, 2003 (182) | USA | AMR | High | 1999-2001 | 2 years | Prison | 1, NA | 7, NA (TST) | NA | unknown | 32 inmates active TB, 96 TST conversions | Inmate | Nosocomial | Unknown |
| Mor et al, 2018 (183) | Israel | EUR | High | 2012 | NA | Aged Care Setting | 2, NA | NA | 2 Caregivers | 28.5, 0% F | No | HCW | Nosocomial | No |
| Okochi, 2005 (184) | Japan | WPR | High | 1997-2002 | ~6 years | Hospital | 3 in first outbreak, 8%, 0 in second outbreak | 13 in first outbreak, 33%, 58 in second outbreak, 34% (TST)* | Doctors, nurses, medical students | NA | 23 patients | Patient (alive and on autopsy) | Nosocomial | NA |
| Oskin et al, 2019 (185) | Russia | EUR | Upper Middle | 1978-2016 | 38 years | Hospital | 136, NA | NA | 54 nurses, 50 doctors, 32 others | 53.7% between 31-50, 73% F | NA | Unknown (several nosocomial cases) | Nosocomial | Unknown |
| Profitt-Henry, 2010 (186) | USA | AMR | High | NA | NA | Hospital | 0, NA | 11, 19% | 5 nurses, 6 AH | NA | NA | Patient | Nosocomial | No |
| Saleiro et al, 2007 (187) | Portugal | EUR | High | 2005 | 3 months | Hospital | 9, 12% | 3, 4% | 8 nurses, 1 doctor | NA, 89% F (active) | NA | Patient | Nosocomial | No |
| Tasaka et al, 2020 (188) | Japan | WPR | High | 2012 | 4 months | Hospital | 1, 2% | 2, 4% (IGRA) | 1 AH (active case) | NA | 13 TB disease, 13 TB infection | Patient | Nosocomial | No |
| Tipple et al, 2004 (189) | USA | AMR | High | 2004 | NA | Hospital | 1, NA | NA | 1 phlebotomist | NA | 5 patients | Patient | Nosocomial | No |
| Unknown, 2004 (190) | USA | AMR | High | NA | NA | Hospital | 1, 0% | 56, 7% | 1 phlebotomist | NA | NA | Patient | Nosocomial | No |
| Yangthara et al, 2021 (191) | Thailand | SEAR | Upper Middle | NA | NA | Hospital | 2, NA | 0, NA | 2 nurses | 24, 100% F | 24 neonates (active TB) | HCW- nurse | Nosocomial | No |
| Zanetti et al, 2012 (192) | Italy | EUR | High | 2010 | 2 months | Laboratory | 0, NA | 15, 33% IGRA conversions, all reverting back to negative | Lab workers | NA | NA | Laboratory sample | Nosocomial | No |

*baseline IGRA or TST not known

Nurses include nursing assistants and midwives

AH allied health, AMR Region of the Americas, EMR Eastern Mediterranean region, EUR European region, F female, HCW health care worker, IGRA interferon gamma release assay, NA not applicable, SEAR South East Asia region, TB tuberculosis, TST tuberculin skin test, USA United States of America, UK United Kingdom, WPRO Western Pacific region

**Supplement 7: Studies describing outbreaks of pertussis in healthcare workers since 2000**

| **Author, year** | **Country** | **Region** | **Income status** | **Year of outbreak** | **Duration** | **Setting** | **# HCW, attack rate** | **Occupation of HCW** | **Median age/Sex of HCW** | **Non HCW cases** | **Index case** | **Origin** | **Affected HCW Vaccinated^** | **Transmission from HCW to patient** |
| --- | --- | --- | --- | --- | --- | --- | --- | --- | --- | --- | --- | --- | --- | --- |
| Alexander et al, 2008 (193) | UK | EUR | High | 2004 | 1 month | Hospital | 1, NA | Nurse | 41, 100% F | 2 neonates | HCW (nurse) | Nosocomial | NA | Yes |
| Al-Murieb et al, 2008 (194) | Australia | WPR | High | 2004 | 3 months | 3 Aged care facilities | 30, 16.7% | NA | NA | 25 residents | HCW (nurse) | Nosocomial | NA | Yes |
| Bassinet et al, 2004 (195) | France | EUR | High | 2000- 2001 | 4 months | Hospital | 15, NA | 9 nurses, 2 doctors, 1 student, 3 others | 40, 80% F | 2 patients | 3 HCW | Nosocomial | 5/15 Yes, 10/15 No | Yes |
| Baugh et al, 2010 (196) | UK | EUR | High | 2008 | 2 months | Hospital | 4, NA | 4 nurses | 43, NA | 2 community members | Community member | Nosocomial | 3/4 Yes, 1/4 No | Yes |
| Boulay et al, 2006 (197) | USA | AMR | High | 2003 | 2 months | Hospital | 10, NA | 6 nurses, 4 unknown | 41, 80% F | NA | HCW - nurse | Nosocomial | NA | No |
| Bryant et al, 2006 (198) | USA | AMR | High | 2003 | 6 weeks | Hospital | 5, NA | 4 nurses, 1 doctor | NA | NA | Infant | Nosocomial | NA | No |
| Bryant et al, 2005 (199) | USA | AMR | High | 2003 | 16 days | Hospital | First outbreak: 5, NA  Second: 8, NA  Third: 1, NA | First: 1 doctor, 4 nurses  Second: 1 doctor, 5 student nurses, 2 others, Third: 1 doctor | NA | First: 1 infant  Second:  1 infant  Third:  1 patient | Linked to Bryant (one of 4 nurses) in first outbreak | Nosocomial | NA | NA |
| Calugar et al, 2006 (200) | USA | AMR | High | 2003 | NA | Hospital | 17, NA | NA | NA | NA | Infant | Nosocomial | NA | No |
| Crameri et al, 2008 (201) | Switzerland | EUR | High | NA | 4 weeks | Hospital | 3, 12% | 3 nurses | 59, NA | NA | HCW - nurse | Nosocomial | 1/3 No, 2/3 NA | No |
| Fischer, 2014 (202) | USA | AMR | High | 2011 | NA | Hospital | 10, NA | NA | NA | NA | Neonate | Nosocomial | NA | Yes |
| Hood et al, 2008 (203) | USA | AMR | High | 2004 | 2 months | Hospital | 1, NA | Nurse | NA | 11 infants | HCW | Nosocomial | 1/1 Yes | Yes |
| Karino et al, 2001 (204) | Japan | WPR | High | 2000 | 4 months | Hospital | 8, NA | NA | 44 | 6 patients | NA | Nosocomial | NA | Yes |
| Leekha et al, 2009 (205) | USA | AMR | High | 2004-2005 | 17 months | Hospital | First outbreak: 13 HCW, NA second outbreak: 64 HCW, NA | Various | NA | First outbreak: 96 cases, Second 58 cases | NA | Frist community, second nosocomial | NA | NA |
| Miyashita et al, 2011 (206) | Japan | WPR | High | 2010 | 4 months | Laboratory | 4, 40% | 4 lab workers | 47.5, 100% F | 5 household contacts | Lab worker | Nosocomial | NA | Yes |
| Nakamuraet al, 2016 (207) | Japan | WPR | High | 2013 - 2014 | 7 months | Outpatient hemodialysis facility | 8, NA | Nurse and other non clinical others | NA | 8 patients | Patient | Nosocomial | NA | NA |
| Petridou et al, 2017 (208) | UK | EUR | High | 2015- 2016 | 2 weeks | Hospital | 1, 2% | 1 midwife | NA | NA | HCW - doctor | Nosocomial | 1/1 No | No |
| Spearing et al, 2002 (209) | Australia | WPR | High | 2001 | 41 days | Hospital | 1, NA | Nurse | NA | NA | Parent of a neonate | Nosocomial | NA | NA |
| Succo et al. 2015 (210) | France | EUR | High | 2013 | 6 weeks | Aged Care facility | 4, 6% | NA | 54, 100% F | 21 residents | HCW, Nurse | Nosocomial | 18% overall vaccinated | NA |
| Vanjak et al, 2006 (211) | France | EUR | High | 2022 | 4 months | Hospital | 10, NA | Nurses, midwives | NA | NA | HCW - 2 midwives | Nosocomial | NA | NA |
| Ward et al, 2005 (212) | France | EUR | High | 2000-2001 | 5 months | Hospital | 15, NA | NA | NA | 2 patients | 3 HCWs | Nosocomial | NA | Yes |
| Yasmin et al, 2012 (213) | USA | AMR | High | 2011 | 2 months | Hospital | 10, NA | NA | 39.5, 60% F | 5 neonates | neonate | Nosocomial | NA | NA |
| Zivna et al, 2007 (214) | USA | AMR | High | 2003-2004 | 1 year | Hospital | 3, 1% | NA | NA | 18 others | 2 HCW, 1 patient (multiple outbreaks) | Community-Acquired | NA | NA |

^prior to outbreak

Nurses include nursing assistants and midwives

AH allied health, AMR Region of the Americas, EMR Eastern Mediterranean region, EUR European region, F female, HCW health care worker, NA not applicable, SEAR South East Asia region, USA United States of America, UK United Kingdom, WPRO Western Pacific region

**Supplement 8: Studies describing outbreaks of rubella in healthcare workers since 2000**

| **Author, year** | **Country** | **Region** | **Income status** | **Year of outbreak** | **Duration** | **Setting** | **# HCW, attack rate** | **Occupation of HCW** | **Median age (years)/Sex of HCW** | **Non HCW cases** | **Index case** | **Origin** | **Affected HCW Vaccinated^** | **Transmission from HCW to patient** |
| --- | --- | --- | --- | --- | --- | --- | --- | --- | --- | --- | --- | --- | --- | --- |
| Singh et al, 2010 (215) | India | SEAR | Lower Middle | 2008 | 6 weeks | Hospital | 23, NA | 2 nurses, 18 medical students, 3 admin | 26, 61% F | 3 residents | HCW- student | Nosocomial | NA | NA |
| Nerome et al, 2004 (216) | Japan | WPR | High | 2004 | 2 months | Hospital | 15, NA | Doctors, nurses, others | 38, 87% F | NA | Patient | Nosocomial | 3/15 Yes, 9/15 No, 3 NA | No |

^prior to outbreak

Nurses include nursing assistants and midwives

F female, HCW health care worker, NA not applicable, SEAR South East Asia region, WPRO Western Pacific region

**Supplement 9: list of included studies in scoping review**

1. Alexander DC, Winter AL, Eshaghi A, Dooling K, Frenette C, De Villa E, et al. Transmission of influenza A pandemic (H1N1) 2009 virus in a long-term care facility in Ontario, Canada. Infect Control Hosp Epidemiol. 2010;31(12):1300–2.

2. Apisarnthanarak A, Mundy LM. Outbreak of influenza A (2009) H1N1 among Thai healthcare workers: is it time to integrate a vaccination program? Infect Control Hosp Epidemiol. 2010;31:854–6.

3. Apisarnthanarak A, Puthavathana P, Kitphati R, Auewarakul P, Mundy LM. Outbreaks of influenza a among nonvaccinated healthcare workers: implications for resource-limited settings. Infect Control Hosp Epidemiol. 2008;29:777–80.

4. Apisarnthanarak A, Mundy LM. Influenza outbreak among health care workers in an avian influenza (H5N1)-endemic setting. Clin Infect Dis. 2006;43(11):1493–4.

5. Aujayeb A, Russell A, Walton K, Samuel J, Waugh S, Valappil M, et al. Influenza: an outbreak in a UK respiratory centre. Br J Nurs. 2013;22(21):1206.

6. Badawi M, Lloyd-Smith E, Leung V, Pincock T, Gustafson R, Romney MG, et al. Management of a Concurrent Influenza A and Parainfluenza 1 Outbreak in a Residential Care Facility. J Am Geriatr Soc. 2016;64(11):e223–5.

7. Balkhy HH, El-Saed A, Sallah M. Epidemiology of H1N1 (2009) influenza among healthcare workers in a tertiary care center in Saudi Arabia: a 6-month surveillance study. Infect Control Hosp Epidemiol. 2010;31:1004–10.

8. Banu A, Sathishchandra H, Anand M. Outbreak of H1N1 influenza among the health care personnel in a tertiary care hospital. J Clin Diagnostic Res. 2012;5(6):1234–6.

9. Bearden A, Friedrich TC, Goldberg TL, Byrne B, Spiegel C, Schult P, et al. An outbreak of the 2009 influenza a (H1N1) virus in a children’s hospital. Influenza Other Respir Viruses. 2012;6(5):374–9.

10. Benet T, Amour S, Valette M, Saadatian-Elahi M, Aho-Glele LS, Berthelot P, et al. Incidence of asymptomatic and symptomatic influenza among healthcare workers: a multicenter prospective cohort study. Clin Infect Dis. 2020;72(9):e311–8.

11. Bhadelia N, Sonti R, McCarthy JW, Vorenkamp J, Jia H, Saiman L, et al. Impact of the 2009 influenza A (H1N1) pandemic on healthcare workers at a tertiary care center in New York City. Infect Control Hosp Epidemiol. 2013;34(8):825–31.

12. Bourlon MT, Macias AE, de la Torre A, Gulias-Herrero A, Leal PE, Dominguez-Cherit G, et al. Organization of a Third-level Care Hospital in Mexico City during the 2009 Influenza Epidemic. Arch Med Res. 2009;40(8):681–6.

13. Bradley-Stewart A, Miller RS, MacLean A, Aitken C, Whittaker L, Gregory V, et al. Cluster of influenza A cases in vaccinated population of adults in Virology Laboratory in Glasgow in December 2012. Scott Med J. 2014;59(2):95–102.

14. Bush KA, McAnulty J, McPhie K, Reynolds R, Boomer M, Clarkson LM, et al. Antiviral prophylaxis in the management of an influenza outbreak in an aged care facility. Commun Dis Intell. 2004;28(3):396–400.

15. Calatayud L, Six C, Duponchel JL, Sillam F, Charlet F, Leussier JJ, et al. Influenza outbreaks in two retirement homes in the Bouches-du-Rhone district, France, March-April 2008. (Special Issue: Infections transmitted from healthcare workers-to-patients.) [French]. Bulletin Epidemiologique Hebdomadaire. 2009;18(19):189–92.

16. Chan MC, Lee N, Ngai KL, Wong BC, Lee MK, Choi KW, et al. A “pre-seasonal” hospital outbreak of influenza pneumonia caused by the drift variant A/Victoria/361/2011-like H3N2 viruses, Hong Kong, 2011. J Clin Virol. 2013;56(3):219–25.

17. Chang YM, Li WC, Huang CT, Huang CG, Tsao KC, Cheng YH, et al. Use of oseltamivir during an outbreak of influenza A in a long-term care facility in Taiwan. J Hosp Infect. 2008;68(1):83–7.

18. Chen LF, Dailey NJ, Rao AK, Fleischauer AT, Greenwald I, Deyde VM, et al. Cluster of oseltamivir-resistant 2009 pandemic influenza A (H1N1) virus infections on a hospital ward among immunocompromised patients--North Carolina, 2009. J Infect Dis. 2011;203(6):838–46.

19. Chen MI, Lee VJ, Barr I, Lin C, Goh R, Lee C, et al. Risk factors for pandemic (H1N1) 2009 virus seroconversion among hospital staff, Singapore. Emerg Infect Dis. 2010;16(10):1554–61.

20. Cheng VC, Tai JW, Wong LM, Chan JF, Li IW, To KK, et al. Prevention of nosocomial transmission of swine-origin pandemic influenza virus A/H1N1 by infection control bundle. J Hosp Infect. 2010;74(3):271–7.

21. Choi S, Chung J, Jeon M, Lee M. Risk factors for pandemic H1N1 2009 infection in healthcare personnel of four general hospitals. J Infect. 2011;63(4):267–73.

22. Chokephaibulkit K, Assanasen S, Apisarnthanarak A, Rongrungruang Y, Kachintorn K, Tuntiwattanapibul Y, et al. Seroprevalence of 2009 H1N1 virus infection and self-reported infection control practices among healthcare professionals following the first outbreak in Bangkok, Thailand. Influenza Other Respir Viruses. 2013;7(3):359–63.

23. Chu TP, Li CC, Wang L, Hsu LW, Eng HL, You HL, et al. A surveillance system to reduce transmission of pandemic H1N1 (2009) influenza in a 2600-bed medical center. PLoS One. 2012;7(3).

24. Considine J, Shaban RZ, Patrick J, Holzhauser K, Aitken P, Clark M, et al. Pandemic (H1N1) 2009 Influenza in Australia: Absenteeism and redeployment of emergency medicine and nursing staff. Emerg Med Australas. 2011;23(5):615–23.

25. Currie AJ, Heslop DJ, Winter SM. H1N1 in the field: The impact on Australian Defence Force Field Exercise Talisman Sabre 09. Aust Emerg Nurs J. 2011;14(2):103–7.

26. de Perio MA, Brueck SE, Mueller CA, Milne CK, Rubin MA, Gundlapalli A V, et al. Evaluation of 2009 pandemic influenza A (H1N1) exposures and illness among physicians in training. Am J Infect Control. 2012;40(7):617–21.

27. Du M, Suo J, Jia N, Xing Y, Xie L, Liu Y. The cross-transmission of 2009 pandemic influenza A (H1N1) infections among healthcare workers and inpatients in a Chinese tertiary hospital. Infect Control Hosp Epidemiol. 2012;33(3):295–8.

28. Eibach D, Casalegno JS, Bouscambert M, Bénet T, Regis C, Comte B, et al. Routes of transmission during a nosocomial influenza A(H3N2) outbreak among geriatric patients and healthcare workers. J Hosp Infect. 2014;86(3):188–93.

29. Fujita M, Matsumoto H, Inafuku Y, Toyama J, Fujita J. A retrospective observational study of the treatment of a nosocomial infection caused by oseltamivir-resistant influenza virus A with baloxavir marboxil. Respir Investig. 2020;58(5):403–8.

30. Fujita J, Tateyama M, Higa F, Nakamatsu M, Owan T, Yamashiro T, et al. Prophylactic oseltamivir for prevention of nosocomial influenza a virus infection. Infect Med. 2008;25(1):49–50a.

31. Gaillat J, Dennetière G, Raffin-Bru E, Valette M, Blanc MC. Summer influenza outbreak in a home for the elderly: application of preventive measures. J Hosp Infect. 2008;70(3):272–7.

32. Gallagher N, Johnston J, Crookshanks H, Nugent C, Irvine N. Characteristics of respiratory outbreaks in care homes during four influenza seasons, 2011-2015. J Hosp Infect. 2018;99(2):175–80.

33. Laris Gonzalez A, Villa Guillen M, Lopez Martinez B, Gamino Arroyo AE, Moreno Espinosa S, Jimenez Juarez RN, et al. Influenza-like illness in healthcare personnel at a paediatric referral hospital: Clinical picture and impact of the disease. Influenza Other Respir Viruses. 2018;12(4):475–81.

34. Grund S, Roggendorf M, Schweiger B. Outbreak of influenza virus A/H1N1 in a hospital ward for immunocompromised patients. Arch Virol. 2010;155(11):1797–802.

35. Guy R, Lambert S, Kelly H. Estimating influenza vaccine effectiveness in an outbreak when anti-viral medications were used as a control measure. Aust N Z J Public Health. 2005;29(6):540–3.

36. Guy RJ, Di Natale R, Kelly HA, Lambert SB, Tobin S, Robinson PM, et al. Influenza outbreaks in aged-care facilities: Staff vaccination and the emerging use of antiviral therapy. Med J Aust. 2004;180(12):640–2.

37. Novel influenza A (H1N1) virus infections in three pregnant women - United States, April-May 2009. MMWR Morb Mortal Wkly Rep. 2009 May;58(18):497–500.

38. Horcajada JP, Pumarola T, Martinez JA, Tapias G, Bayas JM, Prada M de la, et al. A nosocomial outbreak of influenza during a period without influenza epidemic activity. Eur Respir J. 2003;21(2):303–7.

39. Huang ST, Cheng WL. The experience of managing an outbreak of influenza A at a hemodialysis center in a district hospital of Taiwan. Int J Antimicrob Agents. 2017;50:S226.

40. Ishikane M, Kamiya H, Kawabata K, Higashihara M, Sugihara M, Tabuchi A, et al. Seasonal influenza vaccine (A/New York/39/2012) effectiveness against influenza A virus of health care workers in a long term care facility attached with the hospital, Japan, 2014/15: a cohort study. J Infect Chemother. 2016;22(11):777–9.

41. Jaeger JL, Patel M, Dharan N, Hancock K, Meites E, Mattson C, et al. Transmission of 2009 pandemic influenza A (H1N1) virus among healthcare personnel - Southern California, 2009. Infect Control Hosp Epidemiol. 2011;32(12):1149–57.

42. Rao SRJ, Rao MJ, Nandini S, Umapathy BL. Profile of H1N1 infection in a tertiary care center. Indian J Pathol Microbiol. 2011;54(2):323–5.

43. Javaid W, Ehni J, Gonzalez-Reiche AS, Carre..o JM, Hirsch E, Tan J, et al. Real-time investigation of a large nosocomial influenza A outbreak informed by genomic epidemiology. Clin Infect Dis. 2020;73(11):e4375–83.

44. Kay M, Zerr DM, Englund JA, Cadwell BL, Kuypers J, Swenson P, et al. Shedding of pandemic (H1N1) 2009 virus among health care personnel, Seattle, Washington, USA. Emerg Infect Dis. 2011;17(4):639–44.

45. Outbreak of influenza A(H1N1)pdm2009 in a nursing institute dormitory in Kurigram district. HSB. 2012;10(2):16–22.

46. Kiertiburanakul S, Apivanich S, Muntajit T, Sukkra S, Sirinavin S, Leelaudomlipi S, et al. H1N1 2009 influenza among healthcare workers in a tertiary care hospital in Thailand. J Hosp Infection. 2010;74(3):300–2.

47. Kosnik IG, Gregoric EE, Ribic H, Ribnikar M, Orozen K, Lavtizar J, et al. Influenza outbreak in patients and healthcare professionals of the jesenice general hospital in 2017. [Slovene]. Zdravniski Vestnik. 2019;88(11-12):517–28.

48. Lobo RD, Oliveira MS, Garcia CP, Caiaffa Filho HH, Levin AS. Pandemic 2009 H1N1 influenza among health care workers. Am J Infect Control. 2013;41(7):645–7.

49. Magill SS, Black SR, Wise ME, Kallen AJ, Lee SJ, Gardner T, et al. Investigation of an outbreak of 2009 pandemic influenza A virus (H1N1) infections among healthcare personnel in a Chicago hospital. Infect Control Hosp Epidemiol. 2011;32(6):611–5.

50. Mahida N, Clarke M, Jabeen F. Management of an influenza A outbreak on an acute internal medicine ward. J Hosp Infect. 2016;94(2):154–5.

51. Malavaud S, Malavaud B, Sandres K, Durand D, Marty N, Icart J, et al. Nosocomial outbreak of influenza virus A (H3N2) infection in a solid organ transplant department. Transplantation. 2001;72(3):535–7.

52. Masse S, Minodier L, Heuze G, Blanchon T, Capai L, Falchi A. Influenza-like illness outbreaks in nursing homes in Corsica, France, 2014-2015: epidemiological and molecular characterization. Springerplus. 2016;5(1):1338.

53. Matsushita M, Arise K, Morimoto N, Takeuchi S. End-of-season outbreaks of nosocomial influenza caused by waning vaccine immunity. J Infect Prev. 2020;21(3):119–21.

54. Mejia C, Silvestre M, Cazali I, Garcia J, Sanchez R, Garcia L, et al. Large epidemiological influenza a outbreak in a teaching hospital from Guatemala City. ISRN AIDS. 2012;:638042.

55. Miller DR, Christie GL, Molyneaux P, Currie GP. An outbreak of H1N1 influenza in a respiratory unit. Thorax. 2010;65(10):938–9.

56. Oguma T, Saito R, Masaki H, Hoshino K, Zaraket H, Suzuki Y, et al. Molecular characteristics of outbreaks of nosocomial infection with influenza A/H3N2 virus variants. Infect Control Hosp Epidemiol. 2011;32(3):267–75.

57. Ohara M, Tsubokura M, Hosokawa N, Kami M, Mochizuki T. H1N1 influenza A outbreak among young medical staff members who received single dose of non-adjuvanted split-virion 2009 H1N1 vaccine. Hum Vaccin. 2011;7(1):56–7.

58. O’Meara M, Feely E, O’Brien A, Conlon M. Influenza A outbreak in a community hospital. Ir Med J. 2006;99(6).

59. Perez-Padilla R, de la Rosa-Zamboni D, Ponce de Leon S, Hernandez M, Quiñones-Falconi F, Bautista E, et al. Pneumonia and respiratory failure from swine-origin influenza A (H1N1) in Mexico. New Engl J Med. 2009;361(7):680–9.

60. Pryluka D, Lopardo G, Daciuk L, Stecher D, Bonvehi P, Working group for the study of H1N1 infections in health-care workers AS of IDBAA. Severe acute respiratory disease in health-care workers during the influenza H1N1 pandemic in Argentina. J Infect Dev Ctries. 2013;7(1):36–40.

61. Raymond NJ, Berry N, Blackmore TK, Jefferies S, Norton K, Perrin K, et al. Pandemic influenza A(H1N1)2009 in hospital healthcare workers in New Zealand. Infect Control Hosp Epidemiol. 2012;33(2):196–9.

62. Risa KJ, McAndrew JM, Muder RR. Influenza outbreak management on a locked behavioral health unit. Am J Infect Control. 2009;37(1):76–8.

63. Sayers G, Igoe D, Carr M, Cosgrave M, Duffy M, Crowley B, et al. High morbidity and mortality associated with an outbreak of influenza A(H3N2) in a psycho-geriatric facility. Epidemiol Infect. 2013;141(2):357–65.

64. Schulz-Stubner S, Reska M, Schaumann R. Affected healthcare workers during outbreaks: a report from the German consulting center for infection control (BZH) outbreak registry. Infect Control Hosp Epidemiol. 2019;40(1):113–5.

65. Sunagawa S, Fujita J, Iha Y, Tomishima M, Mukatake S, Owan T, et al. Prevention of a nosocomial infection caused by influenza virus a using prophylactic administration of oseltamivir: With review of literatures. Respirology. 2013;4:164.

66. Tennant E, Fletcher S, Kakar S, Najjar Z, Lord H, Clark P, et al. Factors associated with adverse outcomes during influenza outbreaks in aged care facilities. Aust N Z J Public Health. 2020;44(1):65–72.

67. Tsagris V, Nika A, Kyriakou D, Kapetanakis I, Harahousou E, Stripeli F, et al. Influenza A/H1N1/2009 outbreak in a neonatal intensive care unit. J Hosp Infect. 2012;81(1):36–40.

68. Uršič T, Miksić NG, Lusa L, Strle F, Petrovec M. Viral respiratory infections in a nursing home: a six-month prospective study. BMC Infect Dis. 2016;16:637.

69. Vilar-Compte D, Cornejo-Juarez P, Perez-Jimenez C, Cerezo O, Salazar-Rojas E, Joffe PA, et al. An outbreak of influenza among physicians during the first wave of Mexico City’s 2009 H1N1 epidemic. Salud Publica Mex. 2011;53(2):105–6.

70. Vilella A, Serrano B, Marcos MA, Serradesanferm A, Mensa J, Hayes E, et al. Pandemic influenza A(H1N1) outbreak among a group of medical students who traveled to the Dominican Republic. J Travel Med. 2012;19(1):9–14.

71. Severe atypical pneumonia outbreak associated with influenza A(H1N1)pdm09 in Egypt, 2013-2014 season. Weekly Epidemiological Record. 2014;89(16):161–4.

72. Wilson KE, Wood SM, Schaecher KE, Cromwell KB, Godich J, Knapp MH, et al. Nosocomial outbreak of influenza A H3N2 in an inpatient oncology unit related to health care workers presenting to work while ill. Am J Infect Control. 2019;47(6):683–7.

73. Win MK, Chow A, Chen M, Lau YF, Ooi EE, Leo YS. Influenza B Outbreak among influenza-vaccinated welfare home residents in Singapore. Ann Acad Med Singap. 2010;39(6):448–52.

74. Wise ME, De Perio M, Halpin J, Jhung M, Magill S, Black SR, et al. Transmission of pandemic (H1N1) 2009 influenza to healthcare personnel in the United States. Clin Infect Dis. 2011;52:S198-204.

75. Wong B, Lai R, Chan P, Lee N. A hospital outbreak of seasonal influenza involving three health care workers-implications on the optimal choice of respiratory protection. BMC Proceedings Conference: International Conference on Prevention and Infection Control, ICPIC. 2011;5:S6.

76. Zhang Y, Seale H, Yang P, MacIntyre CR, Blackwell B, Tang S, et al. Factors associated with the transmission of pandemic (H1N1) 2009 among hospital healthcare workers in Beijing, China. Influenza Other Respir Viruses. 2013;7(3):466–71.

77. Agut-Busquet E, Gene Tous E, Navarro G, Gonzalez A. Reemergence of measles in vaccinated patients: Report of 6 cases and proposals for prevention. [Spanish]. Emergencias. 2016;28(3):182–4.

78. Augusto GF, Silva A, Pereira N, Fernandes T, Leca A, Valente P, et al. Report of simultaneous measles outbreaks in two different health regions in Portugal, February to May 2017: Lessons learnt and upcoming challenges. Euro Surveill. 2019;24(3).

79. Augusto GF, Cruz D, Silva A, Pereira N, Aguiar B, Leca A, et al. Challenging measles case definition: Three measles outbreaks in three Health Regions of Portugal, February to April 2018. Euro Surveill. 2018;23(28).

80. Barbadoro P, Marigliano A, Di Tondo E, De Paolis M, Martini E, Prospero E, et al. Measles among healthcare workers in a teaching hospital in central Italy. J Occup Health. 2012;54(4):336–9.

81. Baxi R, Mytton OT, Abid M, Maduma-Butshe A, Iyer S, Ephraim A, et al. Outbreak report: nosocomial transmission of measles through an unvaccinated healthcare worker—implications for public health. J Public Health (Bangkok). 2014;36(3):375–81.

82. Beard F, Franklin L, Donohue S, Moran R, Lambert S, Maloney M, et al. Contact tracing of in-flight measles exposures: lessons from an outbreak investigation and case series, Australia, 2010. West Pac Surveill Response J. 2011;2(3):25–33.

83. Berry L, Palmer T, Wells F, Williams E, Sibal B, Timms J. Nosocomial outbreak of measles amongst a highly vaccinated population in an English hospital setting. Infect Prev Pract. 2019;1(2).

84. Biron C, Beaudoux O, Ponge A, Briend-Godet V, Corne F, Tripodi D, et al. Measles in the Nantes Teaching Hospital during the 2008-2009 epidemic. [French]. Med Mal Infect. 2011;41(8):415–23.

85. Bogowicz P, Waller J, Wilson D, Foster K. Consequences of incomplete measles vaccine uptake in healthcare workers during an outbreak in North East England. J Hosp Infect. 2014;86(2):144–6.

86. Botelho-Nevers E, Cassir N, Minodier P, Laporte R, Gautret P, Badiaga S, et al. Measles among healthcare workers: a potential for nosocomial outbreaks. Euro Surveill. 2011;16(2):19764.

87. Burgess CP, Markey P, Skov S, Dowse G. Measles transmission by “fly-in fly-out” workers in Australia. Aust N Z J Public Health. 2013;37(5):423–6.

88. Chen SY, Anderson S, Kutty PK, Lugo F, McDonald M, Rota PA, et al. Health care-associated measles outbreak in the United States after an importation: challenges and economic impact. J Infect Dis. 2011;203(11):1517–25.

89. Cheng VCC, Wong SC, Wong SCY, Sridhar S, Chen JHK, Yip CCY, et al. Measles outbreak from Hong Kong International Airport to the hospital due to secondary vaccine failure in healthcare workers. Infect Control Hosp Epidemiol. 2019;40(12):1407–15.

90. Choi WS, Sniadack DH, Jee Y, Go UY, So JS, Cho H, et al. Outbreak of measles in the Republic of Korea, 2007: importance of nosocomial transmission. J Infect Dis. 2011;204:S483-90.

91. Corbin V, Beytout J, Auclair C, Chambon M, Mouly D, Chamoux A, et al. Shift of the 2009-2011 measles outbreak from children to adults: an observational review at the University Hospital of Clermont-Ferrand, France. Infection. 2013;41(6):1157–61.

92. Cornelissen L, Grammens T, Leenen S, Schirvel C, Hutse V, Demeester R, et al. High number of hospitalisations and non-classical presentations: lessons learned from a measles outbreak in 2017, Belgium. Epidemiol Infect. 2020;148(e35).

93. Currie J, Davies L, McCarthy J, Perry M, Moore C, Cottrell S, et al. Measles outbreak linked to European B3 outbreaks, Wales, United Kingdom, 2017. Euro Surveill. 2017;22(42):17-00673.

94. Davidson N, Andrews R, Riddell M, Leydon J, Lynch P. A measles outbreak among young adults in Victoria, February 2001. Commun Dis Intell Q Rep. 2002;26(2):273–8.

95. De Swart RL, Wertheim-Van Dillen PME, Van Binnendijk RS, Muller CP, Frenkel J, Osterhaus ADME. Measles in a Dutch hospital introduced by an immunocompromised infant from Indonesia infected with a new virus genotype. Lancet. 2000;355(9199):201–2.

96. Dina J, Hamel J, Antona D, Vabret A. Complete genome sequence of a wild-type measles virus isolated during a 2016 winter outbreak in a refugee settlement in Calais, France. Genome Announc. 2017;5(10).

97. Filia A, Bella A, Manso M del, Baggieri M, Magurano F, Rota MC. Ongoing outbreak with well over 4,000 measles cases in Italy from January to end August 2017 - what is making elimination so difficult? Euro Surveill. 2017;22(37).

98. Filia A, Bella A, Cadeddu G, Milia MR, Del Manso M, Rota MC, et al. Extensive Nosocomial Transmission of Measles Originating in Cruise Ship Passenger, Sardinia, Italy, 2014. Emerg Infect Dis. 2015;21(8):1444–6.

99. Fu J, Jiang C, Wang J, Cai R, Cheng W, Shi L, et al. A hospital-associated measles outbreak in health workers in Beijing: implications for measles elimination in China, 2018. Int J Infect Dis. 2019;78:85–92.

100. Garcia Comas L, Ordobas Gavin M, Sanz Moreno JC, Ramos Blazquez B, Rodriguez Baena E, Cordoba Deorador E, et al. Community-wide measles outbreak in the Region of Madrid, Spain, 10 years after the implementation of the Elimination Plan, 2011-2012. Hum Vaccin Immunother. 2017;13(5):1078–83.

101. Comas LG, Gavin MO, Moreno JCS, Garduno IR, Rodriguez MAG, Carbajo MDL, et al. Community outbreak of measles in Madrid (Spain) caused by an imported case. Open Vaccine Journal. 2010;3:48–54.

102. Georgakopoulou T, Horefti E, Vernardaki A, Pogka V, Gkolfinopoulou K, Triantafyllou E, et al. Ongoing measles outbreak in Greece related to the recent European-wide epidemic. Epidemiol Infect. 2018;146(13):1692–8.

103. Georgakopoulou T, Horefti E, Maltezou H, Gkolfinopoulou K, Vernardaki A, Triantafyllou E, et al. Characteristics of the ongoing measles outbreak in Greece in the context of the recent European-wide epidemic and public health measures. Open Forum Infect Dis. 2018;5:S54.

104. George F, Valente J, Augusto GF, Silva AJ, Pereira N, Fernandes T, et al. Measles outbreak after 12 years without endemic transmission, Portugal, February to May 2017. Euro Surveillance. 2017;22(23):30548.

105. Gohil SK, Okubo S, Klish S, Dickey L, Huang SS, Zahn M. Healthcare workers and post-elimination era measles: lessons on acquisition and exposure prevention. Clin Infect Dis. 2016;62(2):166–72.

106. Grammens T, Schirvel C, Leenen S, Shodu N, Hutse V, Costa EM da, et al. Ongoing measles outbreak in Wallonia, Belgium, December 2016 to March 2017: characteristics and challenges. Euro Surveill. 2017;22(17):30524.

107. Grammens T, Maes V, Hutse V, Laisnez V, Schirvel C, Tremerie JM, et al. Different measles outbreaks in Belgium, January to June 2016 - a challenge for public health. Euro Surveill. 2016;21(32):30313.

108. Hospital-associated measles outbreak - Pennsylvania, March-April 2009. MMWR Morb Mortal Wkly Rep. 2012;61(2):30–2.

109. Grgic-Vitek M, Frelih T, Ucakar V, Prosenc K, Tomazic J, Petrovec M, et al. Spotlight on measles 2010: a cluster of measles in a hospital setting in Slovenia, March 2010. Euro Surveillance. 2010;15(20):19573.

110. Guaita Calatrava R, Giner Ferrando E, Bayo Gimeno J, Yuste Munoz L, Saiz Sanchez C, OrtiLucas RM, et al. Measles nosocomial infection outbreak. Boletin Epidemiologico Semanal. 2011;19(16):220–7.

111. Hahné SJM, Lochlainn LMN, van Burgel ND, Kerkhof J, Sane J, Yap KB, et al. Measles Outbreak Among Previously Immunized Healthcare Workers, the Netherlands, 2014. J Infect Dis. 2016;214(12):1980–6.

112. Hebert A, Louis O, Hankenne L, Michel P. Epidemic of measles in the Verviers Area (Belgium): Management and precautions at the reception in the emergency department. [French]. Rev Med Liege. 2017;72(9):406–9.

113. Hiller U, Mankertz A, Koneke N, Wicker S. Hospital outbreak of measles - evaluation and costs of 10 occupational cases among healthcare worker in Germany, February to March 2017. Vaccine. 2019;37(14):1905–9.

114. Huoi C, Benet T, Neuraz A, Mekki Y, Billaud G, Casalegno J, et al. A report on a large measles outbreak in Lyon area, France, 2010-2011. Clin Microbiol Infect. 2012;3:212.

115. Jane M, Torner N, Vidal MJ. Surveillance plan on recent outbreak of measles and rubella in Catalonia, Spain. Rev Esp Salud Publica. 2015;89(4):397–406.

116. Jia H, Ma C, Lu M, Fu J, Rodewald LE, Su Q, et al. Transmission of measles among healthcare Workers in Hospital W, Xinjiang Autonomous Region, China, 2016. BMC Infect Dis. 2018;18:1-N.PAG.

117. Jones G, Haeghebaert S, Merlin B, Antona D, Simon N, Elmouden M, et al. Measles outbreak in a refugee settlement in Calais, France: January to February 2016. Euro Surveill. 2016;21(11):30167.

118. Jones J, Klein R, Popescu S, Rose K, Kretschmer M, Carrigan A, et al. Lack of Measles Transmission to Susceptible Contacts from a Health Care Worker with Probable Secondary Vaccine Failure - Maricopa County, Arizona, 2015. MMWR Morb Mortal Wkly Rep. 2015;64(30):832–3.

119. Kohnen M, Hoffmann P, Frisch C, Charpentier E, Sausy A, Hubschen JM. Diagnostic challenges and pockets of susceptibility identified during a measles outbreak, Luxembourg, 2019. Euro Surveillance. 2021;26(22):2000012.

120. Komitova R, Kunchev A, Mihneva Z. Measles among healthcare workers in Bulgaria. Clinical Microbiology and Infection. 2011;4:S285.

121. Laure B, Jacques F, Mathilde P, Anne-Marie R, Maider C, Jean S, et al. A major regional measles outbreak: Description of hospitalized cases in 2017-2018 at Bordeaux University Hospital, France. Open Forum Infect Dis. 2020;7(9).

122. Lee SS, Ke CM, Cheng MF, Chen JH, Lin LJ, Hung MN, et al. Nosocomial transmission of undetected, imported measles in Taiwan, 2008. Infect Control Hosp Epidemiol. 2009;30(10):1026–8.

123. Maltezou HC, Dedoukou X, Vernardaki A, Katerelos P, Kostea E, Tsiodras S, et al. Measles in healthcare workers during the ongoing epidemic in Greece, 2017-2018. J Hosp Infect. 2018;100: e261–3.

124. Medic S, Petrovic V, Lonearevic G, Kanazir M, Lazarevic IB, Adrovic SR, et al. Epidemiological, clinical and laboratory characteristics of the measles resurgence in the Republic of Serbia in 2014-2015. PLoS One. 2019;14(10).

125. Moghadami M, Afsarkazerooni P, Ebrahimi M, Soltani M, Razmpoor A, Pirasteh E, et al. Measles Outbreak in South of Iran, Where Vaccine Coverage Was High: A Case-Series Study. Iran J Public Health. 2014;43(3):375–80.

126. Monsel G, Rapp C, Duong TA, Farhi D, Bouaziz JD, Meyssonnier V, et al. Measles in adults: An emerging disease not sparing medical staff. [French]. Ann Dermatol Venereol. 2011;138(2):107–10.

127. Nascimento J, Castro RRT, Nascimento J, Knoploch BB, Duque P, Neves MAO. Coinfection of SARS-CoV-2 and Measles morbillivirus in a front-line health worker in Rio de Janeiro, Brasil. Rev Assoc Med Bras. 2020;66(8):1027–9.

128. Orsi A, Butera F, Piazza MF, Schenone S, Canepa P, Caligiuri P, et al. Analysis of a 3-months measles outbreak in western Liguria, Italy: Are hospitals safe and healthcare workers reliable? J Infect Public Health. 2020;13(4):619–24.

129. Porretta A, Quattrone F, Aquino F, Pieve G, Bruni B, Gemignani G, et al. A nosocomial measles outbreak in Italy, February-April 2017. Euro Surveill. 2017;22(33):30597.

130. Ragusa R, Platania A, Cuccia M, Zappala G, Giorgianni G, D’Agati P, et al. Measles and Pregnancy: Immunity and Immunization-What Can Be Learned from Observing Complications during an Epidemic Year. J Pregnancy. 2020:6532868.

131. Rana S, Saavedra-Campos M, Perkins S, Mohammed-Klein R, Wright A, Cordery R, et al. A descriptive analysis of an outbreak of measles and a multilevel mixed-effects analysis of factors associated with case isolation in healthcare settings, London (February–June 2016). Public Health (Elsevier). 2020;183:55–62.

132. Rota JS, Hickman CJ, Sowers SB, Rota PA, Mercader S, Bellini WJ. Two case studies of modified measles in vaccinated physicians exposed to primary measles cases: high risk of infection but low risk of transmission. J Infect Dis. 2011;204:S559-63.

133. Sa Machado R, Perez Duque M, Almeida S, Cruz I, Sottomayor A, Almeida I, et al. Measles outbreak in a tertiary level hospital, Porto, Portugal, 2018: Challenges in the post-elimination era. Euro Surveill. 2018;23(20) :18-00224.

134. Sillam F, Cua E, Faudeux D, Peloux-Petiot F, Negre A, Six C, et al. Investigation of a measles outbreak with nosocomial transmission in Provence-Alpes-Cote d’Azur and in the Principality of Monaco, March-May 2008. What are the lessons learned? (Numero thematique - Rougeole: donnees sur une epidemie en France et en Europe. Bulletin Epidemiologique Hebdomadaire. 2009;39(40):424–6.

135. Six C, De Canecaude JB, Duponchel JL, Lafont E, Decoppet A, Travanut M, et al. Spotlight on measles 2010: Measles outbreak in the provence-alpes-cote d’azur region, france, january to november 2010 - substantial underreporting of cases. Euro Surveill. 2010;15(50):19754.

136. Song K, Lee JM, Lee EJ, Lee BR, Choi JY, Yun J, et al. Control of a nosocomial measles outbreak among previously vaccinated adults in a population with high vaccine coverage: Korea, 2019. Europ J Clin Microbiol Infect Dis. 2022;41(3):455–66.

137. Tafuri S, Germinario C, Rollo M, Prato R. Occupational risk from measles in healthcare personnel: a case report. J Occup Health. 2009;51(1):97–9.

138. Tajima K, Nishimura H, Hongo S, Hazawa M, Saotome-Nakamura AI, Tomiyama K, et al. Estimation of secondary measles transmission from a healthcare worker in a hospital setting. Int J Infect Dis. 2014;24:11–3.

139. Terada K, Niizuma T, Ogita S, Kataoka N, Niki Y. Outbreak of measles in a hospital and measures taken against hospital infection--evidence of cost and benefits. [Japanese]. Kansenshogaku zasshi. 2001;75(6):480–4.

140. Torner N, Ferras J, Curto L, Rebull J, Sol J, Costa J, et al. Measles outbreak related to healthcare transmission. Vacunas. 2021;22(1):20–7.

141. Torner N, Solano R, Rius C, Dominguez A, Surveillance Network Of Catalonia Spain TM. Implication of health care personnel in measles transmission. Hum Vaccin Immunother. 2015;11(1):288–92.

142. Trmal J, Limberkova R. Report on a measles epidemic in the Usti nad Labem Region. [Czech]. Epidemiologie, Mikrobiologie, Imunologie. 2015;64(3):139–45.

143. Trmal J, Kupcova J, Dvorakova L, Vaculikova D, Limberkova R, Slajova I, et al. Measles re-emerging in the Usti region. [Czech]. Epidemiologie, Mikrobiologie, Imunologie. 2014;63(3):154–9.

144. Imported measles cases hit hospitals; prompt diagnosis, HCW records vital. Hosp Infect Control. 2008;35(5):49–54.

145. Vaccinated Healthcare Worker Acquires Measles Amid Outbreak. Hosp Employee Health. 2018;37(8):91–2.

146. Vainio K, Steen TW, Arnesen TM, Ronning K, Anestad G, Dudman S. Measles virus genotyping an important tool in measles outbreak investigation in Norway, 2011. Euro Surveillance. 2012;17(50):20340.

147. Vink JP, Snell LB, Bernard K, Mitchell H, Heathcock RT, Cordery R, et al. Mapping a nosocomial outbreak of measles, coinciding with a period of sustained transmission in South London in 2018. J Hosp Infect. 2020;105(4):747–51.

148. Westgeest AC, de Mooij D, Eger CY, Delfos NM, van der Feltz M, Visser LG, et al. Measles outbreaks - potential threat for health care professionals. Infect Prev Pract. 2020;2(3):100074.

149. Weston KM, Dwyer DE, Ratnamohan M, McPhie K, Chan SW, Branley JM, et al. Nosocomial and community transmission of measles virus genotype D8 imported by a returning traveller from Nepal. Commun Dis Intell. 2006;30(3):358–65.

150. Zhang Z, Zhao Y, Yang L, Lu C, Meng Y, Guan X, et al. Measles outbreak among previously immunized adult healthcare workers, China, 2015. Can J Infect Dis Med Microbiol. 2016;2016:1742530.

151. Zmerli O, Chamieh A, Maasri E, Azar E, Afif C. A challenging modified measles outbreak in vaccinated healthcare providers. Infect Prev Pract. 2021;3(1):100105.

152. Alanazi KH, Bin Saleh GM, Hathout HM, Shiha HR, El Sherbini SG, Al Saqer TA, et al. Investigation of varicella outbreak among residents and healthcare workers in psychiatric hospital- Saudi Arabia. Arch Environ Occup Health. 2020;76(2):116–20.

153. Aly NYA, Al Obaid I, Al-Qulooshi N, Zahed Z. Occupationally related outbreak of chickenpox in an intensive care unit. Med Princ Pract. 2007;16(5):399–401.

154. Apisarnthanarak A, Kitphati R, Tawatsupha P, Thongphubeth K, Apisarnthanarak P, Mundy LM. Outbreak of varicella-zoster virus infection among Thai healthcare workers. Infect Control Hosp Epidemiol. 2007;28(4):430–4.

155. Behrman A, Schmid DS, Crivaro A, Watson B. A cluster of primary varicella cases among healthcare workers with false-positive varicella zoster virus titers. Infect Control Hosp Epidemiol. 2003;24(3):202–6.

156. Bhatti VK, Budhathoki L, Kumar M, Singh G, Nath A, Nair G V. Use of immunization as strategy for outbreak control of varicella zoster in an institutional setting. Med J Armed Forces India. 2014;70(3):220–4.

157. Gunawan S, Linardi P, Tawaluyan K, Mantik MFJ, Veerman AJP. Varicella outbreak in a pediatric oncology ward: the Manado experience. Asian Pac J Cancer Prev. 2010;11(2):289–92.

158. Leung J, Kudish K, Wang C, Moore L, Gacek P, Radford K, et al. A 2009 varicella outbreak in a Connecticut residential facility for adults with intellectual disability. J Infect Dis. 2010;202:1486–91.

159. Lopez AS, Burnett-Hartman A, Nambiar R, Ritz L, Owens P, Loparev VN, et al. Transmission of a newly characterized strain of varicella-zoster virus from a patient with herpes zoster in a long-term-care facility, West Virginia, 2004. J Infect Dis. 2008;197(5):646–53.

160. Park CS, Kim DS, Kim KH. Varicella outbreak in the patients during group therapy: seroprevalence in a healthcare system during breakthrough varicella occurrence. Clin Exp Vaccine Res. 2013;2(2):140–3.

161. Paul N, Jacob ME. An outbreak of cadaver-acquired chickenpox in a health care setting. Clin Infect Dis. 2006;43(5):599–601.

162. Saidel-Odes L, Borer A, Riesenberg K, Frenkel A, Sherlis R, Bouhnick L, et al. An outbreak of varicella in staff nurses exposed to a patient with localized herpes zoster. Scand J Infect Dis. 2010;42(8):620–2.

163. Sarit S, Shruti S, Deepinder C, Chhina RS. Chicken pox outbreak in the Intensive Care Unit of a tertiary care hospital: Lessons learnt the hard way. Indian J Crit Care Med. 2015;19(12):723–5.

164. Sharma R, Goyal K, Bhatia N, Rana V, Singh MP, Bhalla A, et al. Containment of varicella outbreak in intensive care unit of a tertiary level hospital. J Anaesthesiol Clin Pharmacol. 2021;37(2):279–83.

165. Singh H, Pandya KH, Bhatti VK, Lathwal S, Kumar M. Outbreak control of hospital acquired varicella infection amongst health care workers in a tertiary care hospital. Med J Armed Forces India. 2022;78(2):136-9.

166. Sood S. Occupationally related outbreak of chickenpox in hospital staff: A learning experience. J Clinical Diagn Res. 2013;7(10):2294–5.

167. Yang J, Liu J, Xing F, Ye H, Dai G, Liu M, et al. Nosocomial transmission of chickenpox and varicella zoster virus seroprevalence rate amongst healthcare workers in a teaching hospital in China. BMC Infect Dis. 2019;19(1):582.

168. Borgia P, Cambieri A, Chini F, Coltella L, Delogu G, Di Rosa E, et al. Suspected transmission of tuberculosis in a maternity ward from a smear-positive nurse: preliminary results of clinical evaluations and testing of neonates potentially exposed, Rome, Italy, 1 January to 28 July 2011. Euro Surveill. 2011;16(40):19984.

169. Chen TC, Lu PL, Yang CJ, Lin WR, Lin CY, Jou R, et al. Management of a nosocomial outbreak of *Mycobacterium tuberculosis* Beijing/W genotype in Taiwan: An emphasis on case tracing with high-resolution computed tomography. Jpn J Infect Dis. 2010;63(3):199–203.

170. Diel R, Seidler A, Nienhaus A, Rusch-Gerdes S, Niemann S. Occupational risk of tuberculosis transmission in a low incidence area. Respir Res. 2005;6(1):35.

171. Harris TG, Sullivan Meissner J, Proops D. Delay in diagnosis leading to nosocomial transmission of tuberculosis at a New York City health care facility. Am J Infect Control. 2013;41(2):155–60.

172. Hazard R, Enfield KB, Low DJ, Giannetta ET, Sifri CD. Hidden reservoir: an outbreak of tuberculosis in hospital employees with no patient contact. Infect Control Hosp Epidemiol. 2016;37(9):1111–3.

173. Holden KL, Bradley CW, Curran ET, Pollard C, Smith G, Holden E, et al. Unmasking leading to a healthcare worker *Mycobacterium tuberculosis* transmission. J Hosp Infect. 2018;100(4):e226–32.

174. Huang W, Jou R, Yeh P, Angela Huang AT. Laboratory investigation of a nosocomial transmission of tuberculosis at a district general hospital. J Formos Med Assoc. 2007;106(7):520–7.

175. Jonsson J, Kan B, Berggren I, Bruchfeld J. Extensive nosocomial transmission of tuberculosis in a low-incidence country. J Hosp Infect. 2013;83(4):321–6.

176. Kazama H, Nigorikawa H, Kashiwa M, Miyokawa S, Tanaka M, Ichioka M, et al. [Contact investigation using QuantiFERON-TB Gold test to evaluate TB exposure in 61 subjects in a hospital setting--(2) Change in QuantiFERON response during one year after exposure]. [Japanese]. Kekkaku. 2013;88(4):411–6.

177. Khalil NJ, Kryzanowski JA, Mercer NJ, Ellis E, Jamieson F. Tuberculosis outbreak in a long-term care facility. Canadian J Pub Health. 2013;104(1):e28-32.

178. Lai CC, Hsieh YC, Yeh YP, Jou RW, Wang JT, Pan SL, et al. A pulmonary tuberculosis outbreak in a long-term care facility. Epidemiol Infect. 2016;144(7):1455–62.

179. Laniado-Laborin R, Navarro-Alvarez S. Tuberculosis outbreak among health care workers in a general hospital. [Spanish]. Revista del Instituto Nacional de Enfermedades Respiratorias. 2007;20(3):189–94.

180. Luzzati R, Migliori GB, Zignol M, Cirillo DM, Maschio M, Tominz R, et al. Children under 5 years are at risk for tuberculosis after occasional contact with highly contagious patients: Outbreak from a smear-positive healthcare worker. Eur Respir J. 2017;50(5).

181. Malone JL, Ijaz K, Lambert L, Rosencrans L, Phillips L, Tomlinson V, et al. Investigation of healthcare-associated transmission of *Mycobacterium tuberculosis* among patients with malignancies at three hospitals and at a residential facility. Cancer. 2004;101:2713–21.

182. McLaughlin SI, Spradling P, Drociuk D, Ridzon R, Pozsik CJ, Onorato I. Extensive transmission of *Mycobacterium tuberculosis* among congregated, HIV-infected prison inmates in South Carolina, United States. Int J Tuberc Lung Dis. 2003;7(7):665–72.

183. Mor Z, Nuss N, Savion M, Nissan I, Lidji M, Maneshcu S, et al. Tuberculosis outbreak in a nursing home involving undocumented migrants and Israeli citizens. Isr J Health Policy Res. 2018;7(1):36.

184. Okochi Y. Hospital outbreak of Mycobacterium tuberculosis resulting from autopsy exposure. [Japanese]. Kansenshogaku zasshi. 2005;:534–42.

185. Oskin DN, Uryasiev OM, Lunyakov VA, Panfilov YA, Malchuk AP. Disease incidence of occupational tuberculosis in Ryazan region last 30 years. Pakistan Journal of Medical and Health Sciences. 2019;13(2):539–41.

186. Profitt-Henry A. A tuberculosis outbreak in a hospital. Appl Occup Environ Hyg. 2001;16(9):847–8.

187. Saleiro S, Santos AR, Vidal O, Carvalho T, Torres Costa J, Agostinho Marques J. [Tuberculosis in hospital department health care workers]. Rev Port Pneumol. 2007;13(6):789–99.

188. Tasaka M, Koeda E, Takahashi C, Ota M. A tuberculosis outbreak in a psychiatric hospital: Kanagawa, Japan, 2012. Epidemiol Infect. 2019;148:e7.

189. Anonymous. Tuberculosis outbreak in a community hospital--District of Columbia, 2002. MMWR Morb Mortal Wkly Rep. 2004;53(10):214–6.

190. Undiagnosed patient spreads TB to HCW. Hosp Employee Health. 2004.

191. Yangthara B, Wutthigate P, Roongmaitree S, Siripattanapipong P, Lapphra K, Kitsommart R, et al. Nosocomial TB in two neonatal intensive care units at a tertiary care centre: Infection risk and outcomes. Int J Tuberc Lung Dis. 2021;25(7):567–72.

192. Zanetti C, Peracchi M, Zorzi D, Fiorio S, Fallico L, Palu G. Outbreak of transient conversions of the QuantiFERON-TB gold in-tube test in Laboratory health care worker screenings. Clin Vaccine Immunol. 2012;19(6):954–60.

193. Alexander EM, Travis S, Booms C, Kaiser A, Fry NK, Harrison TG, et al. Pertussis outbreak on a neonatal unit: identification of a healthcare worker as the likely source. J Hosp Infect. 2008;69(2):131–4.

194. Al-Murieb A, Brown AM, Raulli A, George C, Gander C, Forrester P, et al. Evidence of pertussis clusters in three aged-care facilities in the former Macquarie Area Health Service, NSW. NSW Public Health Bull. 2008;19(9-10):157–60.

195. Bassinet L, Matrat M, Njamkepo E, Aberrane S, Housset B, Guiso N. Nosocomial pertussis outbreak among adult patients and healthcare workers. Infect Control Hosp Epidemiol. 2004;25(11):995–7.

196. Baugh V, McCarthy N. Outbreak of Bordetella pertussis among oncology nurse specialists. Occup Med (Chic Ill). 2010;60:401–5.

197. Boulay BR, Murray CJ, Ptak J, Kirkland KB, Montero J, Talbot EA. An outbreak of pertussis in a hematology-oncology care unit: implications for adult vaccination policy. Infect Control Hosp Epidemiol. 2006;27(1):92–5.

198. Bryant KA, Humbaugh K, Brothers K, Wright J, Pascual FB, Moran J, et al. Measures to control an outbreak of pertussis in a neonatal intermediate care nursery after exposure to a healthcare worker. Infect Control Hosp Epidemiol. 2006;27(6):541–5.

199. Bryant K, Brothers K, Humbaugh K, Kistler V, Stites S, Madeja S, et al. Outbreaks of Pertussis Associated with Hospitals --- Kentucky, Pennsylvania, and Oregon, 2003. MMWR Morb Mortal Wkly Rep. 2005;54(3):67–71.

200. Calugar A, Ortega-Sanchez IR, Tiwari T, Oakes L, Jahre JA, Murphy T V. Nosocomial pertussis: costs of an outbreak and benefits of vaccinating health care workers. Clin Infect Dis. 2006;42(7):981–8.

201. Crameri S, Heininger U. Successful control of a pertussis outbreak in a university children’s hospital. Int J Infect Dis. 2008;12(6):e85–7.

202. Fischer PR. Pertussis Outbreak in a Newborn ICU costs $100,000 to Contain. Infectious Disease Alert. 2014;33(9):98–9.

203. Anonymous. Hospital-acquired pertussis among newborns -- Texas, 2004. MMWR Morb Mortal Wkly Rep. 2008;57(22):600–3.

204. Karino T, Osaki K, Nakano E, Okimoto N. A pertussis outbreak in a ward for severely retarded. [Japanese]. Kansenshogaku zasshi. 2001;75(11):916–22.

205. Leekha S, Thompson RL, Sampathkumar P. Epidemiology and control of pertussis outbreaks in a tertiary care center and the resource consumption associated with these outbreaks. Infect Control Hosp Epidemiol. 2009;30(5):467–73.

206. Miyashita N, Kawai Y, Yamaguchi T, Ouchi K, Kurose K, Oka M. Outbreak of pertussis in a university laboratory. Intern Med. 2011;50(8):879–85.

207. Nakamura K, Kobayashi M, Yamamoto N, Tokuda K, Miura S, Abe Y, et al. Pertussis outbreak among patients and healthcare workers in a provincial dialysis facility in Japan. J Hosp Infect. 2016;94(4):341–5.

208. Petridou C, Gray H, Heard M, Sugden L, Davis-Blues K, Cortes N, et al. Outbreak of pertussis among healthcare workers in a hospital maternity unit. J Infect Prev. 2017;18(5):253–5.

209. Spearing NM, Horvath RL, McCormack JG. Pertussis: Adults as a source in healthcare settings. Med J Aust. 2002;177(10):568–9.

210. Succo T, Braunstein D, Desmons S, Sampol P, Belchior E, Guiso N, et al. Pertussis outbreak in a nursing home for dependent elderly people, Bouches-du-Rhone (France), August 2013. Bulletin Epidemiologique Hebdomadaire. 2015;5:83–8.

211. Vanjak D, Delaporte MF, Bonmarin I, Levardon M, Fantin B. Cases of pertussis among healthcare workers in a maternity ward: management of a health alert [French]. Med Mal Infect. 2006;36(3):151–6.

212. Ward A, Caro J, Bassinet L, Housset B, O’Brien JA, Guiso N. Health and economic consequences of an outbreak of pertussis among healthcare workers in a hospital in France. Infect Control Hosp Epidemiol. 2005;26(3):288–92.

213. Yasmin S, Sunenshine R, Bisgard KM, Wiedeman C, Carrigan A, Sylvester T, et al. Healthcare-associated pertussis outbreak in Arizona: Challenges and economic impact, 2011. J Pediatric Infect Dis Soc. 2014;3(1):81–4.

214. Zivna I, Bergin D, Casavant J, Fontecchio S, Nelson S, Kelley A, et al. Impact of Bordetella pertussis exposures on a Massachusetts tertiary care medical system. Infect Control Hosp Epidemiol. 2007;28(6):708–12.

215. Singh MP, Diddi K, Dogra S, Suri V, Varma S, Ratho RK. Institutional outbreak of rubella in a healthcare center in Chandigarh, North India. J Med Virol. 2010;82(2):341–4.

216. Nerome Y, Nishi J, Fujiyama R, Takei S, Yoshinaga M, Kawano Y. An outbreak of rubella among hospital personnel and measures taken against hospital infection--cost-benefits of the measure. [Japanese]. Kansenshogaku zasshi. 2004;78(11):967–74.
